# Rapid antibiotic susceptibility testing for urinary tract infections in secondary care in England: a cost-effectiveness analysis

**DOI:** 10.1136/bmjopen-2023-081865

**Published:** 2024-11-28

**Authors:** Ross D Booton, Emily Agnew, Diane Pople, Stephanie Evans, Lucy J Bock, J Mark Sutton, Julie V Robotham, Nichola R Naylor

**Affiliations:** 1 UK Health Security Agency, London, UK; 2 King's College London, London, UK; 3 NIHR Health Protection Research Unit in Healthcare Associated Infections and Antimicrobial Resistance at University of Oxford, Oxford, UK; 4 London School of Hygiene & Tropical Medicine, London, UK

**Keywords:** Urinary tract infections, HEALTH ECONOMICS, Diagnostic microbiology

## Abstract

**ABSTRACT:**

**Objectives:**

To perform a model-based cost-effectiveness evaluation of a rapid antimicrobial susceptibility test.

**Design:**

A Markov model of a cohort of hospital inpatients with urinary tract infection (with inpatient numbers based on national administrative data from 1 April 2017 to 31 March 2019).

**Setting:**

Urinary tract infections (UTI) in acute National Health Service (NHS) Trusts in England, from the perspective of the NHS Healthcare system, at a national level.

**Participants:**

A simulated cohort of approximately 280 000 non-pregnant adult inpatients within secondary care with a clinical suspicion of UTI.

**Interventions:**

Evaluation of the implementation of a fast bacterial impedance cytometry test (BICT) compared with current practice.

**Primary and secondary outcome measures:**

Incremental cost, quality-adjusted life years, net monetary benefit, and bed days and appropriateness of antibiotic use per patient. Costs are presented in 2022 GBP.

**Results:**

Considering benefits arising from reduced time on inappropriate treatment, BICT gives an average net monetary benefit (NMB) over the simulation period of approximately £4.3 million and dominates culture methods (from the healthcare system perspective and with a willingness to pay threshold of £20 000 per quality-adjusted life year). Total inappropriate prescribing days due to the BICT test are reduced by 57%. The extent of the benefit from BICT implementation was strongly dependent on prevalence of resistance, with the NMB increasing sevenfold to over £30 million in a high (40%) resistance prevalence scenario. At the population level, the patient groups with the highest cost and quality-adjusted life year impacts were 65–100-year-old females, followed by males, with uncomplicated UTIs. At an individual patient level, however, 16–64-year-old females with complicated UTIs with oral treatment, followed by 65–100-year-old males with complicated UTIs with oral treatment, were impacted to the greatest degree by the rapid BICT.

**Conclusions:**

Under conservative assumptions and for wide parameter sensitivity, the implementation of BICT would be cost-effective from the NHS healthcare system perspective.

**Strengths and limitations of this study**

- A novel mathematical model is used to quantify both the costs and health benefits of a rapid susceptibility test applied to urinary tract infections (UTIs) in a secondary care setting in England.
- Health impacts are measured in quality-adjusted life years in the analysis, allowing comparisons with other healthcare decisions to be made.
- Appropriate sensitivity analyses are conducted to highlight particular areas of uncertainty in which more information/data would inform cost-effective decision-making.
- We do not present a dynamic transmission model, and therefore, the impact of prevented onward transmission is not captured meaning findings are likely conservative.
- The pathway of UTI patients is complex and simplifying assumptions are made, the model is parameterised by previously published studies and and retrospective data.

## Introduction

 The WHO has declared that antimicrobial resistance (AMR) is one of the biggest health threats facing humanity,^1^ with 300 million deaths and at least a 2% reduction in global gross domestic product expected due to drug resistance in the next 35 years.^2^ The majority of Gram-negative bacterial infections in Europe are caused by *Escherichia coli* (*E. coli*), accounting for the majority of hospital and community-acquired infections,^3^ including around 80% of all urinary tract infections (UTIs).^4^ Hospital admissions due to resistant UTIs are increasing in the UK,^5^ and rates of resistance to antibiotics are increasing.^6^

While UTIs are a major cause of admission to the emergency department and hospitals,^7^ diagnosis is particularly challenging.^8^ Distinguishing UTI from other clinical conditions is difficult. Patients with UTIs typically present with dysuria, pyuria and suprapubic tenderness.^9^ However, they can also present with atypical symptoms.^10^ Patients with a clinical suspicion of UTI in secondary care are generally diagnosed through a positive dipstick test result for nitrate and the presence of leucocytes.^9 11^ In the case of uncomplicated UTI, urine culture is taken, and antibiotics are only given when cultures are reported after 24–72 hours, although antibiotics are likely to be prescribed prior to this if the UTI is recurrent, if the patient has symptoms of pyelonephritis, sepsis or symptoms of complicated UTI or the patient is vulnerable (eg, children, pregnant women or patient over the age of 65).^9^

With diagnostic tests using culture-dependent microbiological samples of blood or urine taking 24–72 hours to return susceptibility profiles,^12^ antibiotics are often prescribed empirically without any culture-indicated results, with no alternate fast antimicrobial susceptibility test (AST) currently widely implemented in hospitals.^12^ This potentially contributes to the wider problem of inappropriate use of antibiotics, which in turn can promote resistance.^13^ An estimated 40%–50% of antibiotic prescriptions for UTIs are unnecessary or inappropriate.^14^

In this context, an impedance-based fast antimicrobial susceptibly test (iFAST) using bacterial impedance cytometry technology (BICT) is under development.^15^ This technique measures changes in electrical and morphological properties of bacteria using microfluidic impedance cytometry, yielding antibiotic sensitivities in less than 5 hours.^15^ Through the timely provision of resistance profiles, the intention is to help inform tailored prescribing decisions, decreasing the time to appropriate and effective therapy, and thereby, improving the management of patients with resistant infections in hospital.^12^ This improved prescribing should improve patient outcomes as well as seeing financial benefits linked to the de-escalation of costly therapy and reduced hospital stays. Further, improved antibiotic stewardship should lead to onward benefits in terms of preventing resistance development in the population more widely.

Previous studies have proven useful in understanding the cost-effectiveness and cost-benefit of implementing interventions or new technologies in healthcare systems, with health economics having wide applications in the field of AMR,^16^,^19^ though a comprehensive exploration of the cost-effectiveness of rapid diagnostics in UTI patients in secondary care at the national level in England has yet to be performed.

We therefore aim to perform a cost-effectiveness assessment of the use of BIC technology such as iFAST in secondary care and investigate whether the technology is deemed economically effective, from the healthcare payer perspective.^20^

## Methods

We developed a cost-effectiveness model and used this to undertake a cost-effectiveness evaluation of the rapid BICT implemented for inpatients with UTI in acute National Health Service (NHS) Trusts in England, from the perspective of the NHS Healthcare system, at a national level (see online supplemental figure 1 for more explanation on perspectives).

### Patient and public involvement

The iFAST Patient Advisory Group, formed as part of the National Institute for Health and Care Research Invention for Innovation (i4i) Programme, were consulted on the health economic evaluation, and, in a bespoke health economics session, input was gained on types of outcomes that the model should evaluate. Out of all measures presented that could be potentially modelled through standard economic evaluation modelling procedures, the advisory group highlighted the importance of including bed days and inappropriate prescribing measures. This led to the inclusion of bed day and time on inappropriate antibiotics being presented within this manuscript. The authors have acknowledged iFAST Patient Advisory Group contributions through the acknowledgement statement. Initial results of this study were presented to the Advisory Group at their regular meeting. Lay summaries of this manuscript will also be disseminated through this group.

### Model structure

**Figure 1:**
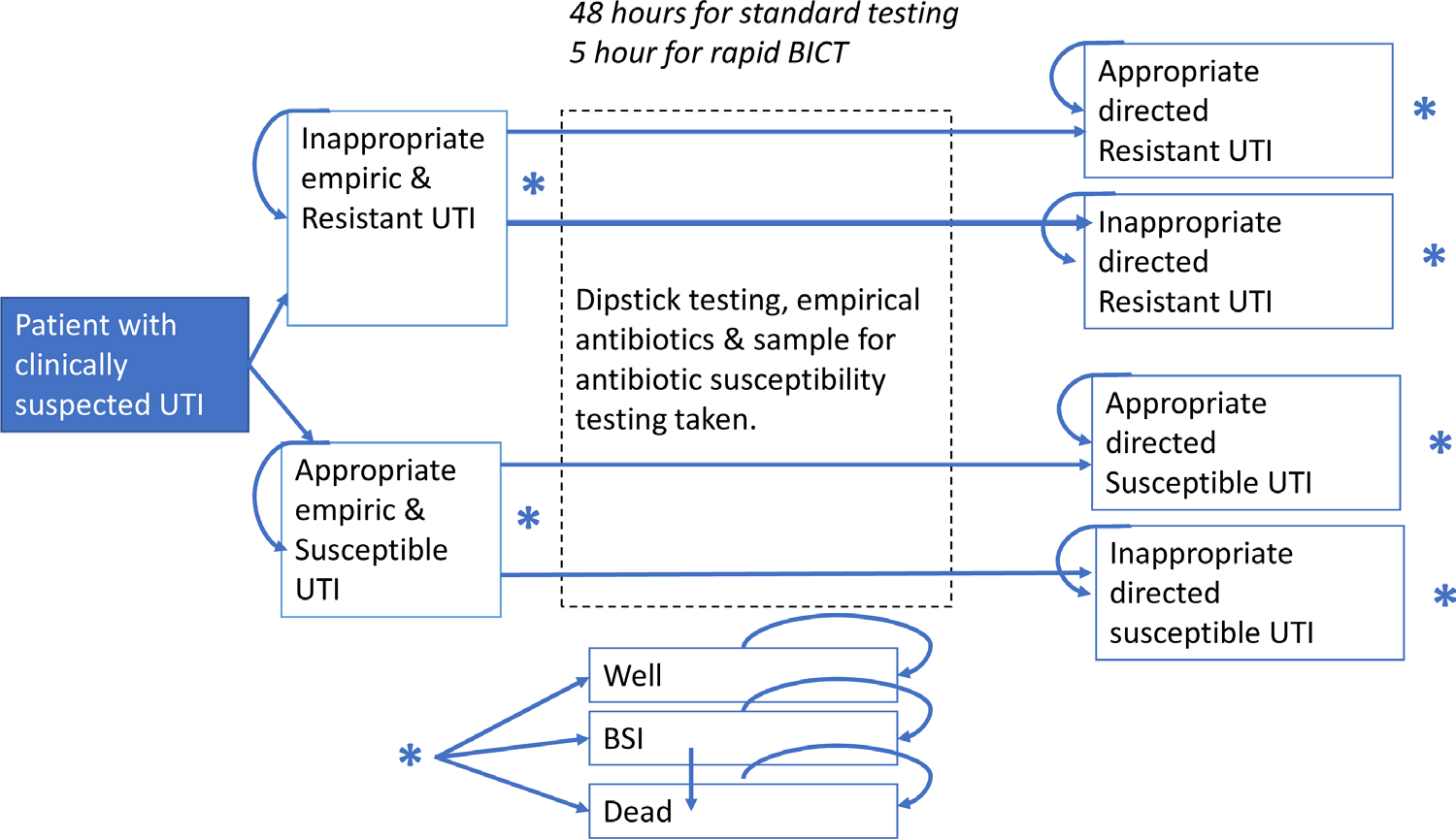
Markov model structure. The patient pathway represents the flow of patients with clinical suspicion of urinary tract infection (UTI) through the health states within hospital. Arrows represent direction of movement; boxes represent health and treatment states. Stars represent potential transitions to well, bloodstream infection (BSI) and death from any of the UTI health states. BICT stands for bacterial impedance cytometry technology.

We developed a Markov model (figure 1) to estimate the potential implications of the BICT test compared with the baseline standard diagnostic and prescribing protocol for UTI patients in hospitals in England. A Markov model is a modelling framework whereby patients flow through specified disease states, where at each time point, they may stay in this state or transition into another. This allowed for the flow of patients between infection (with susceptible and resistance bacteria) and non-infection health states within a hospital to be modelled at hour-long time steps (chosen to account for difference in testing procedures, further explained below). The model was run until all patients had reached the final health states. The clinical setting is assumed to be that of average acute, non-specialist NHS hospital trusts in England. The patient pathway is formulated for adult patients within secondary care with a clinical suspicion of UTI (see figure 1).

Patients with a clinical suspicion of UTI enter the model and move through the Markov model with hourly transition rates. In the baseline model, empirical antibiotics are given to the patient and urine culture is taken, after which the results of the culture take an average of 48 hours (between 24 and 72),^9^ at which point antibiotic choice is reviewed according to susceptibilities. We assume that urine culture and empirical antibiotics occur simultaneously, as the clinical guidelines differ depending on the severity of UTI symptoms and type of UTI.^9^ Patients within the BICT arm of the model move from the empirical prescribing state at a faster rate due to receiving the susceptibility results at 5 hours.^12 15^

The impact of different durations in appropriate or inappropriate treatment states (for resistant infections) is modelled through (i) length of hospital stay, which in turn impacts a patient’s probability of (ii) progression to bloodstream infection (BSI); (iii) admission to intensive care unit (ICU); and (iv) mortality risk. Patients with an untreated UTI develop BSI with a fixed probability at every time point while still in hospital throughout the simulation, as such a longer hospital stay will increase probability of progression to BSI.

All patients in UTI and BSI states have an associated bed-day cost and quality-adjusted life year (QALY) decrement attached, while mortality is associated with a total QALY loss.

The modelled patient cohort is split into age and sex categories on admission; 16–64 years; 65 plus and male; female. These population groups determine antibiotic prescription regimens (empirical and second line). The treatment pathways were built predominantly using National Institute for Health and Care Excellence (NICE) guidelines,^21^,^23^ where more than one pathway was given in the NICE guidelines, other NHS Trust guidelines available online through desk-based review were used.^24 25^ For complicated UTIs, the same guidelines as acute pyelonephritis were used. Children and patients using delivery facilities were excluded from the analysis due to the difference in testing protocols, treatment pathways and associated outcomes.^21^,^23^

Several studies quantify the total complicated/uncomplicated split for patients with UTIs, with values ranging from 9.4% to 22% (in the USA)^26^ and 32% (for males in the Netherlands).^27^ We therefore assume 20% of UTIs are complicated (subsequently 10% intravenous, 10% oral) and 80% uncomplicated (online supplemental table 1). These proportions were assumed to be the same across sex-age groups and resistance prevalence was not dependent on complication status, in the absence of more granular data. Therefore, for example, if there were 17 192 male patients aged between 16 and 64 years with UTI, 1719 were assumed to have complicated UTI receiving intravenous treatment and prescribed intravenous gentamicin empirically, and intravenous co-amoxiclav in the base case scenario second line. Only resistance to empirical antibiotics is considered in the model (and subsequently resistance prevalence and diagnostic sensitivity and specificity are parameterised according to the relevant values for that specific antibiotic).

The cycle length is 1 hour to allow for simulation of the impact of the test and patient transitions. The time horizon of the analysis is set to 10 000 hours (almost 14 months) to ensure the entire cohort of UTI patients progress through the model to either recovery or death. However, we use the lifetime horizon of patients to consider the impact over lifetime of early death. We assume a 3.5% discount rate applied to QALYs over a lifetime horizon. As all other costs and effects are within 1 year, no discount rate is applied. The effectiveness of the BICT technology is assessed in comparison to the baseline using the following indicators: average days on inappropriate antibiotic therapy per patient; average patient length of stay (bed days); bloodstream-infection days and QALYs. We conduct a cost-effectiveness analysis, in which costs are expressed in GBP monetary value in 2022. Costs include those related to diagnostics, empirical antibiotics, culture-directed antibiotics, hospitalisation cost per day, ICU cost per day and the cost of conducting susceptibility tests (from an NHS perspective) (table 1 and online supplemental table 2). Cost-effectiveness is reported as net monetary benefit (NMB) assuming a willingness to pay threshold of £20 000, as there were some instances where the BICT technology was lower in cost and/or higher in QALY gains.

**Table 1:** Key parameters used in model

Parameter description	Base case estimate	Distribution and shape/scale parameters	Sources
Hourly rate of UTI patient developing BSI	3.11×10^−6^	Gammashape=0.3895395scale=34 470.07	BSI 1253total UTI 280462time 60 days^29^- log(1-(1253/280462))/ (60*24)
Sensitivity of culture and of BICT to empirical antibiotics	0.95	UniformMin=0.75Max=1.00	Assumption
Specificity of culture and of BICT to empirical antibiotics	0.85	UniformMin=0.75Max=1.00	Assumption
Cost of standard test (£)	15.67	UniformMin=11.7525Max=19.5875	Cost of urine test £3.85 inflated to £4.98^36 37^+ culture testing (staff and test costs) £8.79 inflated to £10.69. This is a one-off cost incurred at the point of use.
Hourly cost of a general bed day (£)	24.6	UniformMin=18.42Max=30.78	General ward cost per (*excess*) bed day £586.59 (*£351*) average inflated and converted to per hour^35 36^
Hourly cost of an ICU bed day (£)	85.07	UniformMin=63.8Max=106.34	ICU cost per day £1621.16 inflated and converted to per hour^35 36^
Proportion of patients with a complicated UTI in ICU	0.139	UniformMin=0.10425Max=0.17375	^30^
Proportion of patients with an uncomplicated UTI in ICU	0	UniformMin=0Max=0.139	Assume base case equal to 0
Proportion of BSI patients in ICU	0.0795	UniformMin=0.059625Max=0.099375	Average ICU care (%) within 24 hours for non-survivors and survivors^41^

BICTbacterial impedance cytometry testUTIurinary tract infection

We simulate cost-effectiveness of the BICT technology compared with culture diagnostics (labelled as *current* scenarios).

We conduct univariate (varying individual parameters) and probabilistic sensitivity analyses (with 1000 runs) to understand the impact of parameter uncertainty on overall outcomes.

The model is coded and simulated using R v.1.2.5019, using *data.table, ggplot* and *dplyr* packages.^28^

### Input parameters

Epidemiological data, such as number of patients, mortality and length of stay were estimated using national data sources. Other input parameters, such as costs and quality of life outcomes, were not available through such data and were, as such, estimated through the literature. Further description of all sources used is given below and in online supplemental table 2.

### Transition probabilities

The flow of hypothetical patients through both models (baseline and BICT arms) is determined by probabilities taken from the literature (table 1 and online supplemental table 1).

Estimates of total UTI admissions within hospitals in England are estimated from NHS Hospital Episode Statistics Admitted Patient Care (HES APC) data, during 2017–18 and 2018–19 (ie, 1 April 2017 to 31 March 2019), with spells completed on/before 31 March 2020 (eg, up to pandemic-related changes in hospital). We exclude regular day and night attenders and delivery patients. We censor spells based on sex (only include male or female) and method of leaving hospital (clinician, self or tribunal discharge or patient death not stillborn). Age bands were based on age at admission in years (0–15, 16–64, 65–74, 75–84, 85–94, 95+). We use the following definitions for extracting diagnostic data from HES APC:

- Bacteria: includes ‘bacterial … infectious agents’
- UTI: includes ‘infection’ with specified location of upper/lower urinary tract, ‘Urinary tract infection, site not specified’
- Bacteraemia: includes bacteraemia, meningococcal bacteraemia
- Sepsis: includes all diagnoses listed under ‘Sepsis (generalised)’ in ICD10 Alphabetical index to diseases and nature of injury (2016) except those with viral-specific causes or related to maternity/peripartum/paediatric

Transition probabilities from UTI and BSI to discharge and death are estimated using the same hospital administrative data. Transition probabilities regarding resistance vs susceptible UTIs and UTI to BSI rates were determined from the literature,^29^ as was proportion of patients in ICU vs general ward.^30^

Baseline UTI and BSI length of stay (LoS) distributions were used to parameterise average transitions out of the UTI and BSI health states, using time to event analyses. We assume that patients’ risk of discharge and mortality is an average based on data from the entire population (split into UTI and bacteraemia).

The average daily discharge probability was calculated by creating a weighted average for length of stay and dividing by the total population. As the discharge probability included those who had died and those who were discharged alive, the proportion who are discharged is then split into proportion discharged alive and proportion discharged dead. As we did not have mortality rates by day or a specific time-to-follow-up from admission, we assume a constant mortality rate across the period.

To account for differences in discharge rates across the different UTI states, we assume that resistance and inappropriate prescribing is a factor in increased LoS. We assume inappropriate prescribing is around 20%–35%^31 32^ and that this is applicable to the resistant population, so if there was a 5% resistance prevalence rate, and 20% inappropriate prescribing 1.5% ‘resistance and inappropriate prescribing’ prevalence. LoS values and the relevant distribution for resistant and susceptible populations are taken from a large Australian study^33^ and sampled and converted into daily discharge probability. Daily probabilities are converted into hourly probabilities using the appropriate formula.

### Cost and effect unit estimates

Antibiotic cost data were taken from the British National Formulary,^34^ where mean values of price per unit were calculated if multiple were given. A bed-day cost was taken from the literature^35^ and inflated to 2022 prices.^36^ This was a weighted average assumed to be in ICU and unit costs of general bed day and ICU bed day taken from the literature.^35^

We set the cost of implementing the BICT technology to the cost of baseline current test.^36 37^ We do this because the cost of the technology is unknown and calculating a threshold price was not the aim of this study.

### Modelled scenarios

Four different scenarios were modelled to compare the implementation of the BICT technology with conventional culture methods:

1. Base case – population level measures the total projected impact of BICT compared with current baseline values in the NHS (5-hour turnaround time compared with current 48 hours) for a cohort size of approximately 280 000, representing a cohort of UTI patients admitted between 2017 and 2019. The assumed resistance to the empirical treatment is 5% for nitrofurantoin, 9% for gentamicin and 13% for cefalexin.
2. Base case – per patient level is identical to the base case population level but for a cohort size of 1, meaning the calculation of per ‘average-patient’ results. This is to allow the exploration of individual case effects vs population level effects, for example, who is impacted the most rather than what patient groups have the largest number of patients in them.
3. Low trimethoprim resistance measures the impact that BICT could have with a lower resistance to trimethoprim (trimethoprim preferred to other treatments). The assumed resistance to the empirical treatment is 5% for trimethoprim, 9% for gentamicin and 13% for cefalexin (online supplemental table 2).
4. High resistance measures the impact that BICT could have if prevalence of resistance profiles for all treatment is set to a hypothetical 40%.

Online supplemental table 3 shows the cohort sizes used within the model for each UTI type (uncomplicated, complicated intravenous, complicated oral), age group (16–64, 65–100) and sex (male and female). Online supplemental table 1 shows the treatment guidelines (antibiotic and route) used for each UTI group.

We adhere to the CHEERS checklist^38^ in the reporting of this model (see online supplemental table 4).

## Results

### Deterministic analyses

In the baseline resistance scenario, the total inappropriate prescribing days (due to resistance to empirical treatment) under standard culture-based testing were 43 204 (assuming a 48-hour turnaround time). This number decreased with BICT (5-hour turnaround time) to a total of 18 572, a percentage decrease of 57% (online supplemental table 5). These results did not differ for the low trimethoprim scenario; however, for the high prevalence of resistance scenario, the total inappropriate prescribing days were 278 022, which decreased to 119 160 with BICT, a percentage decrease of 57.1%.

In the base scenario, the most bed days saved were in the uncomplicated treatment 65–100-year-old female group (229), followed by uncomplicated treatment 65–100 males (150), complicated oral treatment 65–100 females (143) and complicated oral treatment 65–100 males (93). Online supplemental figure 2 shows the breakdown for bed days saved for all age groups, sex and types of UTIs.

For the alternative resistance level scenarios, at a population level, the incremental cost saving, QALY gain and NMB were the largest in the high resistance prevalence scenario (table 2).

**Table 2:** The incremental cost, incremental QALY gain and net monetary benefit at the population level across tested scenarios

Scenario	Incremental cost (£)	Incremental QALY gain	Net monetary benefit (£)
Base	−606 138	186	4 325 749
Low trimethoprim	−628 342	186	4 347 953
High prevalence	−6 826 894	1175	30 329 429

QALYquality-adjusted life year

The incremental QALY gain was equivalent in the base and low trimethoprim scenario (186). The NMB for the high prevalence scenario was over seven times greater than the base scenario. This can be further illustrated in online supplemental figure 3 where the impact of the high resistance prevalence scenario is always greater in scale than the base and low trimethoprim resistance scenarios for all three outcomes, age groups and UTI group.

When accounting for the per-patient-case effects of each scenario, the NMB was greatest for 16–64 complicated intravenous female patients within the high prevalence scenario (£34.05 per patient, table 3). For the base case scenario, NMB per patient case varied between £2 and £11; for the low trimethoprim scenario, this varied between £2 and £11 also; and for the high prevalence scenario, this range was a much greater £21–£34. When comparing the overall population to the per-patient-case results, overall NMB was greatest in the uncomplicated UTI group for all ages and scenarios (online supplemental figure 3), in contrast to the per-patient-case individual level results where the greatest NMB is within the complicated oral and intravenous patients (table 3).

**Table 3:** Net monetary benefit per patient case across tested scenarios

UTI type and cohort	Net monetary benefit per patient case (£)		
	Base case	Low trimethoprim resistance case	High resistance prevalence
64 F uncomplicated	3.21	3.21	26.66
64 M uncomplicated	2.53	2.53	20.86
65–100 F uncomplicated	2.50	2.51	20.74
65–100 M uncomplicated	2.60	2.60	21.47
16–64 F complicated intravenous	7.53	7.55	**34.05**
16–64 M complicated intravenous	5.89	5.90	26.56
65–100 F complicated intravenous	5.90	5.91	26.65
65–100 M complicated intravenous	6.07	6.08	27.38
16–64 F complicated oral	**10.91**	**10.92**	33.99
16–64 M complicated oral	8.53	8.53	26.51
65–100 F complicated oral	8.55	8.56	26.61
65–100 M complicated oral	8.79	8.79	27.33

UTIurinary tract infection

For the base scenario, the incremental cost was lowest when the cost of the BICT was lowest (online supplemental figure 4). The incremental QALY gain was highest when the turnaround time for BICT was shortest (see online supplemental figure 4 for the tradeoff between cost and turnaround time for BICT). To optimise the NMB, both the turnaround time and cost should be minimised. Online supplemental table 6 shows the full deterministic results for each population group.

### Probabilistic sensitivity analyses

Out of the tested parameters, the test sensitivity, the prevalence of resistance, the impact of resistance and inappropriate antibiotics on mortality and the costs of the current and BICT tests had a large impact on cost-effectiveness (online supplemental figure 5A–C).

**Figure 2:**
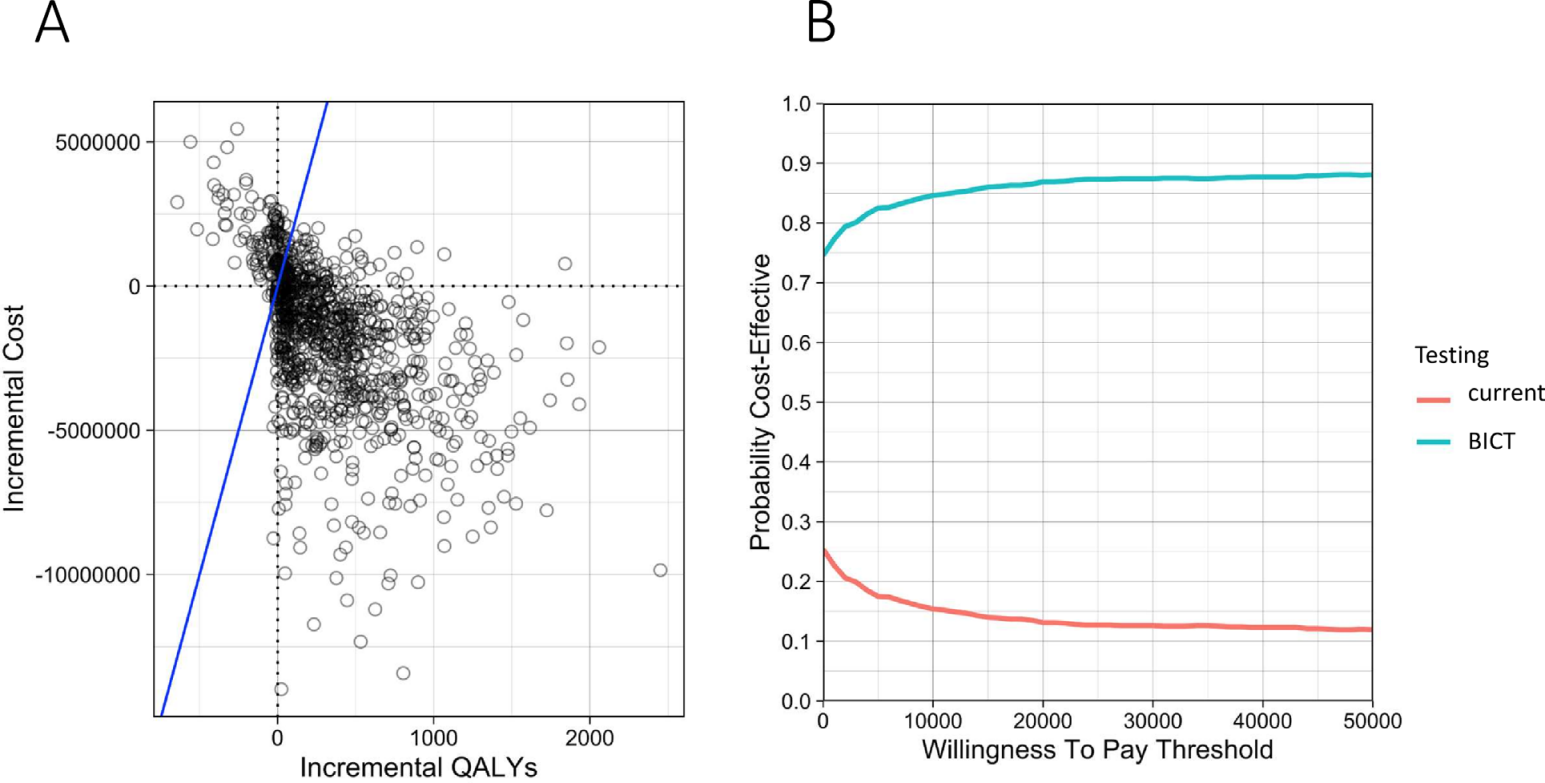
Probabilistic sensitivity analysis results. (**A**) The cost-effectiveness plane. (**B**) The cost-effectiveness acceptability curve.

The model outputs are cost-effective for BICT across willingness-to-pay thresholds—even when accounting for uncertainty in parameters, it is cost-effective (figure 2A). The probabilistic sensitivity analysis (figure 2B) shows that for a wide range of willingness to pay thresholds, the BICT technology is cost-effective.

## Discussion

In this study, we estimate the cost-utility from an NHS system perspective of the BICT. We show that implementing BICT results in NMB (approximately £4.3M in the base case) and dominates current testing practice across a range of willingness to pay for QALYs gained, even when accounting for a high degree of uncertainty in the probabilistic sensitivity analysis.

This is under conservative assumptions, and the impact of the rapid susceptibility test borne out only through reductions in inappropriate prescribing due to resistance. The 57% decrease in inappropriate prescribing days in turn reduces average patient length of stay (by 0.004 days on average per patient), which leads to fewer BSIs, ICU admissions and deaths.

The aim of a rapid diagnostic is to allow tailored treatment. BICT aims to ensure a UTI is treated with an antibiotic to which the causative pathogen is susceptible as rapidly as possible. As such, the effectiveness (and cost-effectiveness) of a rapid AST is determined by the added value of the prescriber knowing these susceptibilities sooner, and this is largely dependent on the prevalence of resistance to the empirical antibiotic choice. In a scenario where resistance prevalence is high, the value a rapid diagnostic brings is greater. This is demonstrated in our model findings, where cost savings, QALY gains and, in turn, NMB, all increased in a higher resistance prevalence scenario. This indicates that it is likely that BICT would become more economical from an NHS healthcare system perspective in a potential future setting of higher resistance to empirical UTI antibiotics.

A strength of this study was the model parameterisation, incorporating a large national dataset (HES APC data to estimate the total UTI admissions within hospitals in England) and consideration of uncertainty distributions for many model parameters. This enables us to predict the likely cost-effectiveness of future implementation of BICT considering parameter uncertainty.

When using these types of models, we must make various assumptions—some of which could be considered weaknesses of this study. For example, while uncertainty was incorporated, this was often with limited information and as such assumed ranges were necessarily chosen for example, plus/minus 25% of values, which might not reflect the true underlying uncertainty of these parameters. Due to inherent complexities in, for example, patient risk factors, comorbidities, history for complicated vs uncomplicated UTI, as well as likely diagnostic work up undertaken, risk of resistance and aetiology, it was necessary to simplify the parameterisation of the pathways and assume parameters (other than treatment regimen) applied across UTI patients. Our data sources for length of stay and mortality are averaged across all UTI patients, which include catheterised patients (who are not included in our model). However, this is counterbalanced as we do not cost other procedures/drugs used for patients in catheterised pathways. Additionally, these data are not disaggregated by pregnancy status; therefore, some of the women in our model would follow different treatment pathways. Delivery patients were removed from the analyses to reduce this potential structural issue. Daily discharge probability for UTI and BSI patients is not adjusted but rather taken from descriptive statistics. Here, total time in hospital should not be misinterpreted as excess time in hospital attributable to UTIs. Additionally, there are not time varying transitions based on previous time in state, as is the limitation with Markov models used in this way, applying a constant probability over time. For the QALY loss at death estimation, we only use age, not gender, and assume that complicated/uncomplicated are the same across different populations (age/sex/resistance status), with the midpoint of age groups used to calculate lost QALYs (which might not be the case in reality if it is mainly those closer to 64 which die). The estimation of the total resistant-inappropriate patients and LoS is taking information from different sources (eg, US-based study). We assume these data are applicable to England, but in the absence of other data, this was necessary and mitigated through our sensitivity analyses showing that BICT is cost-effective for a wide range of parameters.

We did not consider the practicalities of the implementation of BICT for example issues with throughput or additional training required. Capital and implementation costs were not explicitly included and the cost per test was assumed as equal to current practice.

An important note of discussion is that our Markov model does not simulate transmission. If rapid and effective treatment, and additionally changes in patient management, brought about due to the rapid AST led to prevention of transmission, such knock-on cost and health consequences would not be captured. However, these onward impacts, as well as the multiple accompanying externalities across the healthcare economy and more broadly to society, are complex and potentially far reaching. We have therefore necessarily limited scope of this analysis, but robust consideration of wider impacts of diagnostics would benefit from further study.

This results in our cost-effectiveness estimates (table 2) being likely conservative. The mechanism by which bed days are saved is only via a small reduction in average length of stay (0.004 days on average per patient) generated through sooner appropriate treatment for resistant infections (with the impact resistance on UTI itself being conservative). This reduction in average length of stay in turn generates a slightly reduced risk of BSI (due to fewer average days at risk). Furthermore, while we considered implementation of BICT for UTI patients in secondary care, there are obvious potential uses throughout the care pathway. Application of rapid diagnostics in primary care where many UTIs are seen and generate a large burden of antibiotic prescribing (nearly 23% of antibiotic prescriptions in primary care that are linked to a condition are for the urogenital tract^39^) should be assessed in terms of feasibility, effectiveness and cost-effectiveness.

The new UK 5-year National Action Plan for AMR 2024–29^40^ outlines key ways of tackling AMR, including ‘Reducing the need for, and unintentional exposure to antimicrobials’ (Theme 1) and ‘Optimising the use of antimicrobials’ (Theme 2) alongside investing in innovation. Furthermore, specific human health targets are to reduce total antibiotic use in human populations by 5% from the 2019 baseline; prevent any increase in a specified set of drug-resistant infections in humans from the 2019 to 2020 financial year baseline; and prevent any increase in Gram-negative bloodstream infections in humans from the 2019 to 2020 financial year baseline, all by 2029. Therefore, the findings of this study indicate that a rapid BICT such as iFAST could contribute to the goals of the National Action Plan through supporting reductions in inappropriate antibiotic prescribing in secondary care and reduced progression to BSI as well as reducing the development of infections resistant to antibiotics.

## supplementary material

10.1136/bmjopen-2023-081865 online supplemental file 1

## Data Availability

All data relevant to the study are included in the article or uploaded as supplementary information. Hospital Episode Statistics (HES) data were used in this study under license. However, these data are subject to restrictions, are not publicly accessible, and require permission from NHS Digital for access.
