# Supplementary material for: Rapid antibiotic susceptibility testing for urinary tract infections in secondary care in England: a cost-effectiveness analysis
Source: BMJ Open. 2024 Nov 28;14(11):e081865. doi: 10.1136/bmjopen-2023-081865 (PMC11867666; doi:10.1136/bmjopen-2023-081865)
Supplement: online supplemental file 1 [file bmjopen-14-11-s001.pdf]

SUPLEMENTARY MATERIAL

FIGURE 1 Cost-Effectiveness from the Government Perspective vs Budget Impact from the Trust Perspective Introduction Box

- The NHS has a finite set of resources to spend on healthcare interventions. Cost-effectiveness analyses seeks to, at the national level, from the payer perspective, maximise the health gains per pound spent.
- Costs are estimated as the average direct costs per patient flowing through the model, whilst health gains are estimated as the Quality-adjusted life years (QALYs)
- The cost-effectiveness formulae is: (Change in Cost)/(Change in QALYS) < "Willingness to pay threshold"
- This creates the "cost per QALY gained" outcome, we then compare this to a threshold of £20,000 - £30,000 in England. (1)
- When we have something that is both less costly and more effective, with both potentially changing due to scenario/sensitivity analyses, it is important to reorganise the cost-effectiveness formulae in order to not confuse size and sign (positive/negative) when comparing across modelled scenarios. Here we use:
- Net Monetary Benefit (NMB) = (Change in QALYS)*Threshold - (Change in Cost)
- "Cost-effectiveness in a measure of value—how much benefit as achieved for each unit of cost. Affordability is how much you can buy with whatever funds are available. So, cost-effectiveness is independent of any budget, whereas affordability is dependent on a budget." (2)

- This is different from the Trust level budget impact of funding an intervention, as a trust-level budget impact analysis would differ in the calculations in both the costs and effects:
  - Costs could include capital and estate costs of the hospital
  - Effects could include payment (profit/loss) per patients (difference in payment by the government for treating that patient vs actual costs in treating) and would not focus on quality adjusted life years
  - Budget available to actually afford the intervention in question
  - None of these are included in our cost-effectiveness analyses (standard practice for cost-effectiveness analyses of this kind).
  - As budget impact analyses estimate both costs and effects in monetary terms from the perspective of that trust's financial balance sheets.
- Therefore, the results presented should not be taken to be indicative of budget impacts of diagnostic uptake at the trust level, however they can give a conservative estimate of potential differences in "cost per patient" and "outcome per patient" from the government perspective. We say conservative due to the assumptions made and described in the manuscript.

Table 1. Patient Treatment Guidelines Utilised for Non-pregnant Adults

UTI type	Sex	Route	Scenario	First-line Antibiotic	mg	Times per day	days
uncomplicated	women	oral	base	nitrofurantoin	100	2	3
uncomplicated	men	oral	base	nitrofurantoin	100	2	7
uncomplicated	women	oral	low trimethoprim resistance	trimethoprim	200	2	3
uncomplicated	men	oral	low trimethoprim resistance	trimethoprim	200	2	7
complicated	men	IV	base	gentamicin	511	1	7
complicated	women	IV	base	gentamicin	431	1	7
complicated	men	IV	low trimethoprim resistance	gentamicin	511	1	7
complicated	women	IV	low trimethoprim resistance	gentamicin	431	1	7
complicated	women	oral	base	cefalexin	500	2.5	8.5
complicated	men	oral	base	cefalexin	500	2.5	8.5
complicated	women	oral	low trimethoprim resistance	cefalexin	500	2.5	8.5
complicated	men	oral	low trimethoprim resistance	cefalexin	500	2.5	8.5

UTI type	Sex	Route	Scenario	Second-line Antibiotic	mg	Times per day	days
uncomplicated	women	oral	base	pivmecillinam	266.67	3	3
uncomplicated	men	oral	base	co-amoxiclav	500/125	3	8.5
uncomplicated	women	oral	low trimethoprim resistance	nitrofurantion	100	2	3
uncomplicated	men	oral	low trimethoprim resistance	nitrofurantoin	100	2	7
complicated	men	IV	base	co-amoxiclav	1200	1	7
complicated	women	IV	base	co-amoxiclav	1200	1	7
complicated	men	Oral	low trimethoprim resistance	trimethoprim	200	2	14
complicated	women	oral	low trimethoprim resistance	trimethoprim	200	2	14
complicated	women	oral	base	co-amoxiclav	500/125	3	8.5
complicated	men	oral	base	co-amoxiclav	500/125	3	8.5
complicated	women	oral	low trimethoprim resistance	trimethoprim	200	2	14
complicated	Men	oral	low trimethoprim resistance	Trimethoprim	200	2	14

Table 2: Full Parameter Table

*as named in the model, available here: https://github.com/rdbooton/iFAST

UTI group	Age group	Sex group	Scenario	Parameter*	Value	Distribution	Shape	Scale	Description	References & Notes	Antibiotic
all	all	all	all	n.cycles	10000	NA			number of cycles	Assumption	
all	all	all	all	cost.standardtest.U	15.67	uniform	11.75	19.59	cost of conducting dipstick + culture (one off)	(3,4); upper and lower bounds +/- 25% deterministic value	
all	all	all	all	cost.ifast.U	15.67	uniform	11.75	19.59	cost of conducting dipstick + BICT (iFAST) (one off)	(3,4); upper and lower bounds +/- 25% deterministic value	
all	all	all	all	cost.bed.general	24.6	uniform	18.42	30.78	hourly cost of a general bed day	(4,5); daily costs divided by 24 hours	
all	all	all	all	cost.bed.icu	85.07	uniform	63.8	106.34	hourly cost of an icu bed day	(4,5); daily costs divided by 24 hours	
uncomplicated	all	all	all	prop.uti.icu	0	uniform	0	0.139	proportion of uti patients in icu to weight unit costs	Assumption; upper bound set to “complicated” proportion	
complicated_IV	all	all	all	prop.uti.icu	0.139	uniform	0.10425	0.17375	proportion of uti patients in icu to weight unit costs	(6)	
complicated_oral	all	all	all	prop.uti.icu	0.139	uniform	0.10425	0.17375	proportion of uti patients in icu to weight unit costs	(6)	
all	all	all	all	prop.bsi.icu	0.0795	uniform	0.059625	0.099375	proportion of bsi patients in icu	(7)	
all	all	all	all	q.ruti	0.63	beta	129.5527	62.5388	Quality of life for Resistant UTI patients	(8)	
all	all	all	all	q.suti	0.674	beta	147.1142	86.1642	Quality of life for Resistant UTI patients	(8)	
all	all	all	all	q.nouti	0.858	beta	55.6619	9.1594	Quality of life for patients with no UTI	(8)	
all	all	all	all	q.bsi.dec	0.18665	lnormal.95	0.047615	0.279975	Quality of life decrement associated with BSI	(8)	
all	all	all	all	p.uti2bsi	0.000003	gamma	0.38954	34470.07	probability of transitioning to bsi	(9)	
all	all	all	all	t.current	49	NA			hours for current testing results	48 hours +1 (additional 1 hour due to model set up)	
all	all	all	all	t.ifast	6	NA			hours for BICT (iFAST) testing results	5 hours +1 (additional 1 hour due to model set up)	
all	all	all	all	r.multip.los	1.08	lnormal.95	0.95	1.23	multiplier of resistance and inappropriate	Assumption use value for 3GCR vs 3GCS Enterobacteriaceae UTI	

									antibiotics	resistance impact (10)	
all	all	all	all	r.multip.mort	1.3125	uniform	0.984375	1.640625	multiplier of resistance and inappropriate antibiotics	Assumption use value for 3GCR vs 3GCS Enterobacteriaceae UTI resistance impact (10)	
all	all	all	all	prop.inap	0.27	uniform	0.2	0.35	Proportion of UTI patients on inappropriate therapy (used to calculate impact of resistance + being on inappropriate therapy on LOS and mortality)	(11,12)	
all	1664	f	all	qaly.death	16.85	NA			qaly loss from death over the lifetime	(13) note age at death was set to the midpoint of the group (i.e. for 16 – 64 age at death was set to 40), the model referenced was used to calculated QALYs.	
all	1664	f	all	prop.bsi2die	0.07271	uniform	0.054533	0.090888	proportion of bsi discharges which are dead	Calculated from HES data (14). Unadjusted proportions used.	
all	1664	f	all	prop.uti2die	0.025395	uniform	0.019046	0.031744	proportion of uti discharges which are dead	Calculated from HES data (14). Unadjusted proportions used.	
all	1664	f	all	p.uti2discharge_other	0.07347	uniform	0.055102	0.091837	Daily risk of discharge for UTI patients	Calculated from HES data (14).	
all	1664	f	all	p.bsi2discharge	0.0469	uniform	0.035175	0.058625	Daily risk of discharge for BSI patients	Calculated from HES data (14)	
all	1664	m	all	qaly.death	16.85				QALY loss from death over the lifetime	(13) note age at death was set to the midpoint of the group (i.e. for 16 – 64 age at death was set to 40)	
all	1664	m	all	prop.bsi2die	0.085547	uniform	0.06416	0.106933	Proportion of bsi patients discharges that are to death	Calculated from HES data (14)	
all	1664	m	all	prop.uti2die	0.035249	uniform	0.026437	0.044061	Proportion of uti patient discharges that are to death	Calculated from HES data (14)	
all	1664	m	all	p.uti2discharge_other	0.046807	uniform	0.035105	0.058509	Daily UTI to discharge rate	Calculated from HES data (14)	
all	1664	m	all	p.bsi2discharge	0.038128	uniform	0.028596	0.047661	Daily BSI to discharge rate	Calculated from HES data (14)	
all	65100	f	all	qaly.death	5.77				QALY death rate	(13) note age at death was set to the midpoint of the group (i.e. for 16 – 64 age at death was set to 40)	

all	65100	f	all	prop.bsi2die	0.181615	uniform	0.136212	0.227019	Daily BSI to death rate	Calculated from HES data (14)	
all	65100	f	all	prop.uti2die	0.077426	uniform	0.058069	0.096782	Daily UTI to death rate	Calculated from HES data (14)	
all	65100	f	all	p.uti2discharge_other	0.049753	uniform	0.037315	0.062192	Daily UTI to discharge rate	Calculated from HES data (14)	
all	65100	f	all	p.bsi2discharge	0.043627	uniform	0.03272	0.054534	Daily BSI to discharge rate	Calculated from HES data (14)	
all	65100	m	all	qaly.death	5.77				QALY death rate	(13) note age at death was set to the midpoint of the group (i.e. for 16 – 64 age at death was set to 40)	
all	65100	m	all	prop.bsi2die	0.169638	uniform	0.127229	0.212048	Proportion of BSI patients that die	Calculated from HES data (14)	
all	65100	m	all	prop.uti2die	0.09626	uniform	0.072195	0.120324	Proportion of UTI patients that die	Calculated from HES data (14)	
all	65100	m	all	p.uti2discharge_other	0.049562	uniform	0.037172	0.061953	Proportion of UTI patients that are dsicharged	Calculated from HES data (14)	
all	65100	m	all	p.bsi2discharge	0.043289	uniform	0.032467	0.054112	Proportion of BSI patients that are discharged	Calculated from HES data (14)	
uncomplicated	1664	f	all	cohort	25768.8				Total in that cohort	Calculated from HES data (14)	
uncomplicated	1664	f	Base case	r.prev	0.05	uniform	0.02	0.25	prevalence of that resistance profile	Assumption. Estimates in (15–19)	nitrofurantoin
uncomplicated	1664	f	Base case	sensitivity.i	0.95	uniform	0.75	1	sensitivity to that antibiotic – BICT (iFAST)	Assumption	nitrofurantoin
uncomplicated	1664	f	Base case	specificity.i	0.85	uniform	0.75	1	specificity to that antibiotic - BICT (iFAST)	Assumption	nitrofurantoin
uncomplicated	1664	f	Base case	sensitivity.c	0.95	uniform	0.75	1	sensitivity to that antibiotic - current	Assumption	nitrofurantoin
uncomplicated	1664	f	Base case	specificity.c	0.85	uniform	0.75	1	specificity to that antibiotic - current	Assumption	nitrofurantoin
uncomplicated	1664	f	Base case	cost.empiricuti.U	2.15	uniform	0.77	5.79	cost of empiric therapy (one off)	British National Formulary (20)<br>Cost per tab into cost per course (21–25)	nitrofurantoin
uncomplicated	1664	f	Base case	cost.empiricuti.current	1.43	uniform	0.51	3.86	cost of empiric therapy for those that then switch	British National Formulary (20)<br>Cost per tab into cost per course (21–25)	nitrofurantoin
uncomplicated	1664	f	Base case	cost.empiricuti.ifast	0.36	uniform	0.13	0.97	cost of empiric therapy for those that then switch	British National Formulary (20)<br>Cost per tab into cost per course (21–25)	nitrofurantoin
uncomplicated	1664	f	Base case	cost.2ndline.U	6.48	uniform	6.48	9.46	cost of second line (one off)	British National Formulary (20)	pivmecillinam

										Cost per tab into cost per course (21–25)	
complicated_IV	1664	f	all	cohort	3221.1				Total in that cohort	Calculated from HES data (14)	
complicated_IV	1664	f	Base case	r.prev	0.09	uniform	0.069	0.11	prevalence of that resistance profile	Assumption. Estimates in (16,18)	gentamicin
complicated_IV	1664	f	Base case	sensitivity.i	0.95	uniform	0.75	1	sensitivity to that antibiotic - BICT (iFAST)	Assumption	gentamicin
complicated_IV	1664	f	Base case	specificity.i	0.85	uniform	0.75	1	specificity to that antibiotic - BICT (iFAST)	Assumption	gentamicin
complicated_IV	1664	f	Base case	sensitivity.c	0.95	uniform	0.75	1	sensitivity to that antibiotic - current	Assumption	gentamicin
complicated_IV	1664	f	Base case	specificity.c	0.85	uniform	0.75	1	specificity to that antibiotic - current	Assumption	gentamicin
complicated_IV	1664	f	Base case	cost.empiricuti.U	51.89	uniform	45.26	51.89	cost of empiric therapy (one off)	British National Formulary (20)<br>Cost per tab into cost per course (21–25)	gentamicin
complicated_IV	1664	f	Base case	cost.empiricuti.current	14.83	uniform	12.93	14.83	cost of empiric therapy for those that then switch	British National Formulary (20)<br>Cost per tab into cost per course (21–25)	gentamicin
complicated_IV	1664	f	Base case	cost.empiricuti.ifast	5.58	uniform	2.67	5.58	cost of empiric therapy for those that then switch	British National Formulary (20)<br>Cost per tab into cost per course (21–25)	gentamicin
complicated_IV	1664	f	Base case	cost.2ndline.U	20.02	uniform	7.42	35	cost of second line (one off)	British National Formulary (20)<br>Cost per tab into cost per course (21–25)	co-amoxiclav
uncomplicated	65100	f	all	cohort	95461.6				Total in that cohort	Calculated from HES data (14)	
uncomplicated	65100	f	Base case	r.prev	0.05	uniform	0.02	0.25	prevalence of that resistance profile	Assumption. Estimates in (15–19)	nitrofurantoin
uncomplicated	65100	f	Base case	sensitivity.i	0.95	uniform	0.75	1	sensitivity to that antibiotic - BICT (iFAST)	Assumption	nitrofurantoin
uncomplicated	65100	f	Base case	specificity.i	0.85	uniform	0.75	1	specificity to that antibiotic - BICT (iFAST)	Assumption	nitrofurantoin
uncomplicated	65100	f	Base case	sensitivity.c	0.95	uniform	0.75	1	sensitivity to that antibiotic - current	Assumption	nitrofurantoin
uncomplicated	65100	f	Base case	specificity.c	0.85	uniform	0.75	1	specificity to that antibiotic - current	Assumption	nitrofurantoin
uncomplicated	65100	f	Base case	cost.empiricuti.U	2.15	uniform	0.77	5.79	cost of empiric therapy (one off)	British National Formulary (20)<br>Cost per tab into cost per course (21–25)	nitrofurantoin
uncomplicated	65100	f	Base case	cost.empiricuti.current	1.43	uniform	0.51	3.86	cost of empiric therapy for those that then	British National Formulary (20)	nitrofurantoin

									switch	Cost per tab into cost per course (21–25)	
uncomplicated	65100	f	Base case	cost.empiricuti.ifast	0.36	uniform	0.13	0.97	cost of empiric therapy for those that then switch	British National Formulary (20) Cost per tab into cost per course (21–25)	nitrofurantoin
uncomplicated	65100	f	Base case	cost.2ndline.U	6.48	uniform	6.48	9.46	cost of second line (one off)	British National Formulary (20) Cost per tab into cost per course (21–25)	pivmecillinam
complicated_IV	65100	f	all	cohort	11932.7				Total in that cohort	Calculated from HES data (14)	
complicated_IV	65100	f	Base case	r.prev	0.09	uniform	0.069	0.11	prevalence of that resistance profile	Assumption. Estimates in (16,18)	gentamicin
complicated_IV	65100	f	Base case	sensitivity.i	0.95	uniform	0.75	1	sensitivity to that antibiotic - BICT (iFAST)	Assumption	gentamicin
complicated_IV	65100	f	Base case	specificity.i	0.85	uniform	0.75	1	specificity to that antibiotic - BICT (iFAST)	Assumption	gentamicin
complicated_IV	65100	f	Base case	sensitivity.c	0.95	uniform	0.75	1	sensitivity to that antibiotic - current	Assumption	gentamicin
complicated_IV	65100	f	Base case	specificity.c	0.85	uniform	0.75	1	specificity to that antibiotic - current	Assumption	gentamicin
complicated_IV	65100	f	Base case	cost.empiricuti.U	51.89	uniform	45.26	51.89	cost of empiric therapy (one off)	British National Formulary (20) Cost per tab into cost per course (21–25)	gentamicin
complicated_IV	65100	f	Base case	cost.empiricuti.current	14.83	uniform	12.93	14.83	cost of empiric therapy for those that then switch	British National Formulary (20) Cost per tab into cost per course (21–25)	gentamicin
complicated_IV	65100	f	Base case	cost.empiricuti.ifast	5.58	uniform	2.67	5.58	cost of empiric therapy for those that then switch	British National Formulary (20) Cost per tab into cost per course (21–25)	gentamicin
complicated_IV	65100	f	Base case	cost.2ndline.U	20.02	uniform	7.42	35	cost of second line (one off)	British National Formulary (20) Cost per tab into cost per course (21–25)	co-amoxiclav
uncomplicated	1664	m	all	cohort	13753.6				Total in that cohort	Calculated from HES data (14)	
uncomplicated	1664	m	Base case	r.prev	0.05	uniform	0.02	0.25	prevalence of that resistance profile	Assumption. Estimates in (15–19)	nitrofurantoin
uncomplicated	1664	m	Base case	sensitivity.i	0.95	uniform	0.75	1	sensitivity to that antibiotic - BICT (iFAST)	Assumption	nitrofurantoin
uncomplicated	1664	m	Base case	specificity.i	0.85	uniform	0.75	1	specificity to that antibiotic - BICT (iFAST)	Assumption	nitrofurantoin
uncomplicated	1664	m	Base case	sensitivity.c	0.95	uniform	0.75	1	sensitivity to that antibiotic - current	Assumption	nitrofurantoin

uncomplicated	1664	m	Base case	specificity.c	0.85	uniform	0.75	1	specificity to that antibiotic - current	Assumption	nitrofurantoin
uncomplicated	1664	m	Base case	cost.empiricuti.U	5.02	uniform	1.79	13.52	cost of empiric therapy (one off)	British National Formulary (20)<br>Cost per tab into cost per course (21–25)	nitrofurantoin
uncomplicated	1664	m	Base case	cost.empiricuti.current	1.43	uniform	0.51	3.86	cost of empiric therapy for those that then switch	British National Formulary (20)<br>Cost per tab into cost per course (21–25)	nitrofurantoin
uncomplicated	1664	m	Base case	cost.empiricuti.ifast	0.36	uniform	0.13	0.97	cost of empiric therapy for those that then switch	British National Formulary (20)<br>Cost per tab into cost per course (21–25)	nitrofurantoin
uncomplicated	1664	m	Base case	cost.2ndline.U	9.59	uniform	2	14.57	cost of second line (one off)	British National Formulary (20)<br>Cost per tab into cost per course (21–25)	co-amoxiclav
complicated_IV	1664	m	all	cohort	1719.2				Total in that cohort	Calculated from HES data (14)	
complicated_IV	1664	m	Base case	r.prev	0.09	uniform	0.069	0.11	prevalence of that resistance profile	Assumption. Estimates in (16,18)	gentamicin
complicated_IV	1664	m	Base case	sensitivity.i	0.95	uniform	0.75	1	sensitivity to that antibiotic - BICT (iFAST)	Assumption	gentamicin
complicated_IV	1664	m	Base case	specificity.i	0.85	uniform	0.75	1	specificity to that antibiotic - BICT (iFAST)	Assumption	gentamicin
complicated_IV	1664	m	Base case	sensitivity.c	0.95	uniform	0.75	1	sensitivity to that antibiotic - current	Assumption	gentamicin
complicated_IV	1664	m	Base case	specificity.c	0.85	uniform	0.75	1	specificity to that antibiotic - current	Assumption	gentamicin
complicated_IV	1664	m	Base case	cost.empiricuti.U	61.52	uniform	53.66	61.52	cost of empiric therapy (one off)	British National Formulary (20)<br>Cost per tab into cost per course (21–25)	gentamicin
complicated_IV	1664	m	Base case	cost.empiricuti.current	17.58	uniform	15.33	17.58	cost of empiric therapy for those that then switch	British National Formulary (20)<br>Cost per tab into cost per course (21–25)	gentamicin
complicated_IV	1664	m	Base case	cost.empiricuti.ifast	7.68	uniform	3.2	7.68	cost of empiric therapy for those that then switch	British National Formulary (20)<br>Cost per tab into cost per course (21–25)	gentamicin
complicated_IV	1664	m	Base case	cost.2ndline.U	20.02	uniform	7.42	35	cost of second line (one off)	British National Formulary (20)<br>Cost per tab into cost per course (21–25)	co-amoxiclav
uncomplicated	65100	m	all	cohort	62132				Total in that cohort	Calculated from HES data (14)	

uncomplicated	65100	m	Base case	r.prev	0.05	uniform	0.02	0.25	prevalence of that resistance profile	Assumption. Estimates in (15–19)	nitrofurantoin
uncomplicated	65100	m	Base case	sensitivity.i	0.95	uniform	0.75	1	sensitivity to that antibiotic - BICT (iFAST)	Assumption	nitrofurantoin
uncomplicated	65100	m	Base case	specificity.i	0.85	uniform	0.75	1	specificity to that antibiotic - BICT (iFAST)	Assumption	nitrofurantoin
uncomplicated	65100	m	Base case	sensitivity.c	0.95	uniform	0.75	1	sensitivity to that antibiotic - current	Assumption	nitrofurantoin
uncomplicated	65100	m	Base case	specificity.c	0.85	uniform	0.75	1	specificity to that antibiotic - current	Assumption	nitrofurantoin
uncomplicated	65100	m	Base case	cost.empiricuti.U	5.02	uniform	1.79	13.52	cost of empiric therapy (one off)	British National Formulary (20)<br>Cost per tab into cost per course (21–25)	nitrofurantoin
uncomplicated	65100	m	Base case	cost.empiricuti.current	1.43	uniform	0.51	3.86	cost of empiric therapy for those that then switch	British National Formulary (20)<br>Cost per tab into cost per course (21–25)	nitrofurantoin
uncomplicated	65100	m	Base case	cost.empiricuti.ifast	0.36	uniform	0.13	0.97	cost of empiric therapy for those that then switch	British National Formulary (20)<br>Cost per tab into cost per course (21–25)	nitrofurantoin
uncomplicated	65100	m	Base case	cost.2ndline.U	9.59	uniform	2	14.57	cost of second line (one off)	British National Formulary (20)<br>Cost per tab into cost per course (21–25)	co-amoxiclav
complicated_IV	65100	m	all	cohort	7766.5				Total in that cohort	Calculated from HES data (14)	
complicated_IV	65100	m	Base case	r.prev	0.09	uniform	0.069	0.11	prevalence of that resistance profile	Assumption. Estimates in (16,18)	gentamicin
complicated_IV	65100	m	Base case	sensitivity.i	0.95	uniform	0.75	1	sensitivity to that antibiotic - BICT (iFAST)	Assumption	gentamicin
complicated_IV	65100	m	Base case	specificity.i	0.85	uniform	0.75	1	specificity to that antibiotic - BICT (iFAST)	Assumption	gentamicin
complicated_IV	65100	m	Base case	sensitivity.c	0.95	uniform	0.75	1	sensitivity to that antibiotic - current	Assumption	gentamicin
complicated_IV	65100	m	Base case	specificity.c	0.85	uniform	0.75	1	specificity to that antibiotic - current	Assumption	gentamicin
complicated_IV	65100	m	Base case	cost.empiricuti.U	61.52	uniform	53.66	61.52	cost of empiric therapy (one off)	British National Formulary (20)<br>Cost per tab into cost per course (21–25)	gentamicin
complicated_IV	65100	m	Base case	cost.empiricuti.current	17.58	uniform	15.33	17.58	cost of empiric therapy for those that then switch	British National Formulary (20)<br>Cost per tab into cost per course (21–25)	gentamicin
complicated_IV	65100	m	Base case	cost.empiricuti.ifast	7.68	uniform	3.2	7.68	cost of empiric therapy for those that then	British National Formulary (20)	gentamicin

									switch	Cost per tab into cost per course (21–25)	
complicated_IV	65100	m	Base case	cost.2ndline.U	20.02	uniform	7.42	35	cost of second line (one off)	British National Formulary (20)<br>Cost per tab into cost per course (21–25)	co-amoxiclav
complicated_oral	1664	f	Base case	r.prev	0.13	uniform	0.08	0.18	prevalence of that resistance profile	Assumption. Estimates in (16,18)	cefalexin
complicated_oral	1664	f	Base case	sensitivity.i	0.95	uniform	0.75	1	sensitivity to that antibiotic - BICT (iFAST)	Assumption	cefalexin
complicated_oral	1664	f	Base case	specificity.i	0.85	uniform	0.75	1	specificity to that antibiotic - BICT (iFAST)	Assumption	cefalexin
complicated_oral	1664	f	Base case	sensitivity.c	0.95	uniform	0.75	1	sensitivity to that antibiotic - current	Assumption	cefalexin
complicated_oral	1664	f	Base case	specificity.c	0.85	uniform	0.75	1	specificity to that antibiotic - current	Assumption	cefalexin
complicated_oral	1664	f	Base case	cost.empiricuti.U	2.41	uniform	1.49	3.19	cost of empiric therapy (one off)	British National Formulary (20)<br>Cost per tab into cost per course (21–25)	cefalexin
complicated_oral	1664	f	Base case	cost.empiricuti.current	0.57	uniform	0.35	0.75	cost of empiric therapy for those that then switch	British National Formulary (20)<br>Cost per tab into cost per course (21–25)	cefalexin
complicated_oral	1664	f	Base case	cost.empiricuti.ifast	0.11	uniform	0.07	0.15	cost of empiric therapy for those that then switch	British National Formulary (20)<br>Cost per tab into cost per course (21–25)	cefalexin
complicated_oral	1664	f	Base case	cost.2ndline.U	9.59	uniform	2	14.57	cost of second line (one off)	British National Formulary (20)<br>Cost per tab into cost per course (21–25)	co-amoxiclav
complicated_oral	65100	f	Base case	r.prev	0.13	uniform	0.08	0.18	prevalence of that resistance profile	Assumption. Estimates in (16,18)	cefalexin
complicated_oral	65100	f	Base case	sensitivity.i	0.95	uniform	0.75	1	sensitivity to that antibiotic - BICT (iFAST)	Assumption	cefalexin
complicated_oral	65100	f	Base case	specificity.i	0.85	uniform	0.75	1	specificity to that antibiotic - BICT (iFAST)	Assumption	cefalexin
complicated_oral	65100	f	Base case	sensitivity.c	0.95	uniform	0.75	1	sensitivity to that antibiotic - current	Assumption	cefalexin
complicated_oral	65100	f	Base case	specificity.c	0.85	uniform	0.75	1	specificity to that antibiotic - current	Assumption	cefalexin
complicated_oral	65100	f	Base case	cost.empiricuti.U	2.41	uniform	1.49	3.19	cost of empiric therapy (one off)	British National Formulary (20)<br>Cost per tab into cost per course (21–25)	cefalexin
complicated_oral	65100	f	Base case	cost.empiricuti.current	0.57	uniform	0.35	0.75	cost of empiric therapy for those that then	British National Formulary (20)	cefalexin

									switch	Cost per tab into cost per course (21–25)	
complicated_oral	65100	f	Base case	cost.empiricuti.ifast	0.11	uniform	0.07	0.15	cost of empiric therapy for those that then switch	British National Formulary (20) Cost per tab into cost per course (21–25)	cefalexin
complicated_oral	65100	f	Base case	cost.2ndline.U	9.59	uniform	2	14.57	cost of second line (one off)	British National Formulary (20) Cost per tab into cost per course (21–25)	co-amoxiclav
complicated_oral	1664	m	Base case	r.prev	0.13	uniform	0.08	0.18	prevalence of that resistance profile	Assumption. Estimates in (16,18)	cefalexin
complicated_oral	1664	m	Base case	sensitivity.i	0.95	uniform	0.75	1	sensitivity to that antibiotic - BICT (iFAST)	Assumption	cefalexin
complicated_oral	1664	m	Base case	specificity.i	0.85	uniform	0.75	1	specificity to that antibiotic - BICT (iFAST)	Assumption	cefalexin
complicated_oral	1664	m	Base case	sensitivity.c	0.95	uniform	0.75	1	sensitivity to that antibiotic - current	Assumption	cefalexin
complicated_oral	1664	m	Base case	specificity.c	0.85	uniform	0.75	1	specificity to that antibiotic - current	Assumption	cefalexin
complicated_oral	1664	m	Base case	cost.empiricuti.U	2.41	uniform	1.49	3.19	cost of empiric therapy (one off)	British National Formulary (20) Cost per tab into cost per course (21–25)	cefalexin
complicated_oral	1664	m	Base case	cost.empiricuti.current	0.57	uniform	0.35	0.75	cost of empiric therapy for those that then switch	British National Formulary (20) Cost per tab into cost per course (21–25)	cefalexin
complicated_oral	1664	m	Base case	cost.empiricuti.ifast	0.11	uniform	0.07	0.15	cost of empiric therapy for those that then switch	British National Formulary (20) Cost per tab into cost per course (21–25)	cefalexin
complicated_oral	1664	m	Base case	cost.2ndline.U	9.59	uniform	2	14.57	cost of second line (one off)	British National Formulary (20) Cost per tab into cost per course (21–25)	co-amoxiclav
complicated_oral	65100	m	Base case	r.prev	0.13	uniform	0.08	0.18	prevalence of that resistance profile	Assumption. Estimates in (16,18)	cefalexin
complicated_oral	65100	m	Base case	sensitivity.i	0.95	uniform	0.75	1	sensitivity to that antibiotic - BICT (iFAST)	Assumption	cefalexin
complicated_oral	65100	m	Base case	specificity.i	0.85	uniform	0.75	1	specificity to that antibiotic - BICT (iFAST)	Assumption	cefalexin
complicated_oral	65100	m	Base case	sensitivity.c	0.95	uniform	0.75	1	sensitivity to that antibiotic - current	Assumption	cefalexin
complicated_oral	65100	m	Base case	specificity.c	0.85	uniform	0.75	1	specificity to that antibiotic - current	Assumption	cefalexin
complicated_oral	65100	m	Base case	cost.empiricuti.U	2.41	uniform	1.49	3.19	cost of empiric therapy (one off)	British National Formulary (20)	cefalexin

										Cost per tab into cost per course (21–25)	
complicated_oral	65100	m	Base case	cost.empiricuti.current	0.57	uniform	0.35	0.75	cost of empiric therapy for those that then switch	British National Formulary (20)<br>Cost per tab into cost per course (21–25)	cefalexin
complicated_oral	65100	m	Base case	cost.empiricuti.ifast	0.11	uniform	0.07	0.15	cost of empiric therapy for those that then switch	British National Formulary (20)<br>Cost per tab into cost per course (21–25)	cefalexin
complicated_oral	65100	m	Base case	cost.2ndline.U	9.59	uniform	2	14.57	cost of second line (one off)	British National Formulary (20)<br>Cost per tab into cost per course (21–25)	co-amoxiclav
uncomplicated	1664	f	Low trimethoprim resistance	r.prev	0.05	uniform	0.02	0.25	prevalence of that resistance profile	Assumption. Estimates in (15–19)	trimethoprim
uncomplicated	1664	f	Low trimethoprim resistance	sensitivity.i	0.95	uniform	0.75	1	sensitivity to that antibiotic - BICT (iFAST)	Assumption	trimethoprim
uncomplicated	1664	f	Low trimethoprim resistance	specificity.i	0.85	uniform	0.75	1	specificity to that antibiotic - BICT (iFAST)	Assumption	trimethoprim
uncomplicated	1664	f	Low trimethoprim resistance	sensitivity.c	0.95	uniform	0.75	1	sensitivity to that antibiotic - current	Assumption	trimethoprim
uncomplicated	1664	f	Low trimethoprim resistance	specificity.c	0.85	uniform	0.75	1	specificity to that antibiotic - current	Assumption	trimethoprim
uncomplicated	1664	f	Low trimethoprim resistance	cost.empiricuti.U	0.87	uniform	0.71	2.9	cost of empiric therapy (one off)	British National Formulary (20)<br>Cost per tab into cost per course (21–25)	trimethoprim
uncomplicated	1664	f	Low trimethoprim resistance	cost.empiricuti.current	0.58	uniform	0.47	1.93	cost of empiric therapy for those that then switch	British National Formulary (20)<br>Cost per tab into cost per course (21–25)	trimethoprim
uncomplicated	1664	f	Low trimethoprim resistance	cost.empiricuti.ifast	0.15	uniform	0.12	0.48	cost of empiric therapy for those that then switch	British National Formulary (20)<br>Cost per tab into cost per course (21–25)	trimethoprim
uncomplicated	1664	f	Low trimethoprim resistance	cost.2ndline.U	2.15	uniform	0.77	5.79	cost of second line (one off)	British National Formulary (20)<br>Cost per tab into cost per course (21–25)	nitrofurantoin
complicated_IV	1664	f	Low trimethoprim resistance	r.prev	0.09	uniform	0.069	0.11	prevalence of that resistance profile	Assumption. Estimates in (16,18)	gentamicin

complicated_IV	1664	f	Low trimethoprim resistance	sensitivity.i	0.95	uniform	0.75	1	sensitivity to that antibiotic - BICT (iFAST)	Assumption	gentamicin
complicated_IV	1664	f	Low trimethoprim resistance	specificity.i	0.85	uniform	0.75	1	specificity to that antibiotic - BICT (iFAST)	Assumption	gentamicin
complicated_IV	1664	f	Low trimethoprim resistance	sensitivity.c	0.95	uniform	0.75	1	sensitivity to that antibiotic - current	Assumption	gentamicin
complicated_IV	1664	f	Low trimethoprim resistance	specificity.c	0.85	uniform	0.75	1	specificity to that antibiotic - current	Assumption	gentamicin
complicated_IV	1664	f	Low trimethoprim resistance	cost.empiricuti.U	51.89	uniform	45.26	51.89	cost of empiric therapy (one off)	British National Formulary (20)<br>Cost per tab into cost per course (21–25)	gentamicin
complicated_IV	1664	f	Low trimethoprim resistance	cost.empiricuti.current	14.83	uniform	12.93	14.83	cost of empiric therapy for those that then switch	British National Formulary (20)<br>Cost per tab into cost per course (21–25)	gentamicin
complicated_IV	1664	f	Low trimethoprim resistance	cost.empiricuti.ifast	5.58	uniform	2.67	5.58	cost of empiric therapy for those that then switch	British National Formulary (20)<br>Cost per tab into cost per course (21–25)	gentamicin
complicated_IV	1664	f	Low trimethoprim resistance	cost.2ndline.U	4.06	uniform	3.32	13.53	cost of second line (one off)	British National Formulary (20)<br>Cost per tab into cost per course (21–25)	trimethoprim
uncomplicated	65100	f	Low trimethoprim resistance	r.prev	0.05	uniform	0.02	0.25	prevalence of that resistance profile	Assumption. Estimates in (15–19)	trimethoprim
uncomplicated	65100	f	Low trimethoprim resistance	sensitivity.i	0.95	uniform	0.75	1	sensitivity to that antibiotic - BICT (iFAST)	Assumption	trimethoprim
uncomplicated	65100	f	Low trimethoprim resistance	specificity.i	0.85	uniform	0.75	1	specificity to that antibiotic - BICT (iFAST)	Assumption	trimethoprim
uncomplicated	65100	f	Low trimethoprim resistance	sensitivity.c	0.95	uniform	0.75	1	sensitivity to that antibiotic - current	Assumption	trimethoprim
uncomplicated	65100	f	Low trimethoprim resistance	specificity.c	0.85	uniform	0.75	1	specificity to that antibiotic - current	Assumption	trimethoprim
uncomplicated	65100	f	Low trimethoprim resistance	cost.empiricuti.U	0.87	uniform	0.71	2.9	cost of empiric therapy (one off)	British National Formulary (20)<br>Cost per tab into cost per course (21–25)	trimethoprim

uncomplicated	65100	f	Low trimethoprim resistance	cost.empiricuti.current	0.58	uniform	0.47	1.93	cost of empiric therapy for those that then switch	British National Formulary (20) Cost per tab into cost per course (21–25)	trimethoprim
uncomplicated	65100	f	Low trimethoprim resistance	cost.empiricuti.ifast	0.15	uniform	0.12	0.48	cost of empiric therapy for those that then switch	British National Formulary (20) Cost per tab into cost per course (21–25)	trimethoprim
uncomplicated	65100	f	Low trimethoprim resistance	cost.2ndline.U	2.15	uniform	0.77	5.79	cost of second line (one off)	British National Formulary (20) Cost per tab into cost per course (21–25)	nitrofurantoin
complicated_IV	65100	f	Low trimethoprim resistance	r.prev	0.09	uniform	0.069	0.11	prevalence of that resistance profile	Assumption. Estimates in (16,18)	gentamicin
complicated_IV	65100	f	Low trimethoprim resistance	sensitivity.i	0.95	uniform	0.75	1	sensitivity to that antibiotic - BICT (iFAST)	Assumption	gentamicin
complicated_IV	65100	f	Low trimethoprim resistance	specificity.i	0.85	uniform	0.75	1	specificity to that antibiotic - BICT (iFAST)	Assumption	gentamicin
complicated_IV	65100	f	Low trimethoprim resistance	sensitivity.c	0.95	uniform	0.75	1	sensitivity to that antibiotic - current	Assumption	gentamicin
complicated_IV	65100	f	Low trimethoprim resistance	specificity.c	0.85	uniform	0.75	1	specificity to that antibiotic - current	Assumption	gentamicin
complicated_IV	65100	f	Low trimethoprim resistance	cost.empiricuti.U	51.89	uniform	45.26	51.89	cost of empiric therapy (one off)	British National Formulary (20) Cost per tab into cost per course (21–25)	gentamicin
complicated_IV	65100	f	Low trimethoprim resistance	cost.empiricuti.current	14.83	uniform	12.93	14.83	cost of empiric therapy for those that then switch	British National Formulary (20) Cost per tab into cost per course (21–25)	gentamicin
complicated_IV	65100	f	Low trimethoprim resistance	cost.empiricuti.ifast	5.58	uniform	2.67	5.58	cost of empiric therapy for those that then switch	British National Formulary (20) Cost per tab into cost per course (21–25)	gentamicin
complicated_IV	65100	f	Low trimethoprim resistance	cost.2ndline.U	4.06	uniform	3.32	13.53	cost of second line (one off)	British National Formulary (20) Cost per tab into cost per course (21–25)	trimethoprim
uncomplicated	1664	m	Low trimethoprim resistance	r.prev	0.05	uniform	0.02	0.25	prevalence of that resistance profile	Assumption. Estimates in (15–19)	trimethoprim
uncomplicated	1664	m	Low trimethoprim	sensitivity.i	0.95	uniform	0.75	1	sensitivity to that antibiotic - BICT (iFAST)	Assumption	trimethoprim

			resistance								
uncomplicated	1664	m	Low trimethoprim resistance	specificity.i	0.85	uniform	0.75	1	specificity to that antibiotic - BICT (iFAST)	Assumption	trimethoprim
uncomplicated	1664	m	Low trimethoprim resistance	sensitivity.c	0.95	uniform	0.75	1	sensitivity to that antibiotic - current	Assumption	trimethoprim
uncomplicated	1664	m	Low trimethoprim resistance	specificity.c	0.85	uniform	0.75	1	specificity to that antibiotic - current	Assumption	trimethoprim
uncomplicated	1664	m	Low trimethoprim resistance	cost.empiricuti.U	2.03	uniform	1.66	6.77	cost of empiric therapy (one off)	British National Formulary (20) Cost per tab into cost per course (21–25)	trimethoprim
uncomplicated	1664	m	Low trimethoprim resistance	cost.empiricuti.current	0.58	uniform	0.47	1.93	cost of empiric therapy for those that then switch	British National Formulary (20) Cost per tab into cost per course (21–25)	trimethoprim
uncomplicated	1664	m	Low trimethoprim resistance	cost.empiricuti.ifast	0.15	uniform	0.12	0.48	cost of empiric therapy for those that then switch	British National Formulary (20) Cost per tab into cost per course (21–25)	trimethoprim
uncomplicated	1664	m	Low trimethoprim resistance	cost.2ndline.U	5.02	uniform	1.79	13.52	cost of second line (one off)	British National Formulary (20) Cost per tab into cost per course (21–25)	nitrofurantoin
complicated_IV	1664	m	Low trimethoprim resistance	r.prev	0.09	uniform	0.069	0.11	prevalence of that resistance profile	Assumption. Estimates in (16,18)	gentamicin
complicated_IV	1664	m	Low trimethoprim resistance	sensitivity.i	0.95	uniform	0.75	1	sensitivity to that antibiotic - BICT (iFAST)	Assumption	gentamicin
complicated_IV	1664	m	Low trimethoprim resistance	specificity.i	0.85	uniform	0.75	1	specificity to that antibiotic - BICT (iFAST)	Assumption	gentamicin
complicated_IV	1664	m	Low trimethoprim resistance	sensitivity.c	0.95	uniform	0.75	1	sensitivity to that antibiotic - current	Assumption	gentamicin
complicated_IV	1664	m	Low trimethoprim resistance	specificity.c	0.85	uniform	0.75	1	specificity to that antibiotic - current	Assumption	gentamicin
complicated_IV	1664	m	Low trimethoprim resistance	cost.empiricuti.U	61.52	uniform	53.66	61.52	cost of empiric therapy (one off)	British National Formulary (20) Cost per tab into cost per course (21–25)	gentamicin
complicated_IV	1664	m	Low trimethoprim	cost.empiricuti.current	17.58	uniform	15.33	17.58	cost of empiric therapy for those that then	British National Formulary (20)	gentamicin

			resistance						switch	Cost per tab into cost per course (21–25)	
complicated_IV	1664	m	Low trimethoprim resistance	cost.empiricuti.ifast	7.68	uniform	3.2	7.68	cost of empiric therapy for those that then switch	British National Formulary (20)<br>Cost per tab into cost per course (21–25)	gentamicin
complicated_IV	1664	m	Low trimethoprim resistance	cost.2ndline.U	4.06	uniform	3.32	13.53	cost of second line (one off)	British National Formulary (20)<br>Cost per tab into cost per course (21–25)	trimethoprim
uncomplicated	65100	m	Low trimethoprim resistance	r.prev	0.05	uniform	0.02	0.25	prevalence of that resistance profile	Assumption. Estimates in (15–19)	trimethoprim
uncomplicated	65100	m	Low trimethoprim resistance	sensitivity.i	0.95	uniform	0.75	1	sensitivity to that antibiotic - BICT (iFAST)	Assumption	trimethoprim
uncomplicated	65100	m	Low trimethoprim resistance	specificity.i	0.85	uniform	0.75	1	specificity to that antibiotic - BICT (iFAST)	Assumption	trimethoprim
uncomplicated	65100	m	Low trimethoprim resistance	sensitivity.c	0.95	uniform	0.75	1	sensitivity to that antibiotic - current	Assumption	trimethoprim
uncomplicated	65100	m	Low trimethoprim resistance	specificity.c	0.85	uniform	0.75	1	specificity to that antibiotic - current	Assumption	trimethoprim
uncomplicated	65100	m	Low trimethoprim resistance	cost.empiricuti.U	2.03	uniform	1.66	6.77	cost of empiric therapy (one off)	British National Formulary (20)<br>Cost per tab into cost per course (21–25)	trimethoprim
uncomplicated	65100	m	Low trimethoprim resistance	cost.empiricuti.current	0.58	uniform	0.47	1.93	cost of empiric therapy for those that then switch	British National Formulary (20)<br>Cost per tab into cost per course (21–25)	trimethoprim
uncomplicated	65100	m	Low trimethoprim resistance	cost.empiricuti.ifast	0.15	uniform	0.12	0.48	cost of empiric therapy for those that then switch	British National Formulary (20)<br>Cost per tab into cost per course (21–25)	trimethoprim
uncomplicated	65100	m	Low trimethoprim resistance	cost.2ndline.U	5.02	uniform	1.79	13.52	cost of second line (one off)	British National Formulary (20)<br>Cost per tab into cost per course (21–25)	nitrofurantoin
complicated_IV	65100	m	Low trimethoprim resistance	r.prev	0.09	uniform	0.069	0.11	prevalence of that resistance profile	Assumption. Estimates in (16,18)	gentamicin
complicated_IV	65100	m	Low trimethoprim resistance	sensitivity.i	0.95	uniform	0.75	1	sensitivity to that antibiotic - BICT (iFAST)	Assumption	gentamicin
complicated_IV	65100	m	Low	specificity.i	0.85	uniform	0.75	1	specificity to that	Assumption	gentamicin

			trimethoprim resistance						antibiotic - BICT (iFAST)		
complicated_IV	65100	m	Low trimethoprim resistance	sensitivity.c	0.95	uniform	0.75	1	sensitivity to that antibiotic - current	Assumption	gentamicin
complicated_IV	65100	m	Low trimethoprim resistance	specificity.c	0.85	uniform	0.75	1	specificity to that antibiotic - current	Assumption	gentamicin
complicated_IV	65100	m	Low trimethoprim resistance	cost.empiricuti.U	61.52	uniform	53.66	61.52	cost of empiric therapy (one off)	British National Formulary (20) Cost per tab into cost per course (21–25)	gentamicin
complicated_IV	65100	m	Low trimethoprim resistance	cost.empiricuti.current	17.58	uniform	15.33	17.58	cost of empiric therapy for those that then switch	British National Formulary (20) Cost per tab into cost per course (21–25)	gentamicin
complicated_IV	65100	m	Low trimethoprim resistance	cost.empiricuti.ifast	7.68	uniform	3.2	7.68	cost of empiric therapy for those that then switch	British National Formulary (20) Cost per tab into cost per course (21–25)	gentamicin
complicated_IV	65100	m	Low trimethoprim resistance	cost.2ndline.U	4.06	uniform	3.32	13.53	cost of second line (one off)	British National Formulary (20) Cost per tab into cost per course (21–25)	trimethoprim
complicated_oral	1664	f	Low trimethoprim resistance	r.prev	0.13	uniform	0.08	0.18	prevalence of that resistance profile	Assumption. Estimates in (16,18)	cefalexin
complicated_oral	1664	f	Low trimethoprim resistance	sensitivity.i	0.95	uniform	0.75	1	sensitivity to that antibiotic - BICT (iFAST)	Assumption	cefalexin
complicated_oral	1664	f	Low trimethoprim resistance	specificity.i	0.85	uniform	0.75	1	specificity to that antibiotic - BICT (iFAST)	Assumption	cefalexin
complicated_oral	1664	f	Low trimethoprim resistance	sensitivity.c	0.95	uniform	0.75	1	sensitivity to that antibiotic - current	Assumption	cefalexin
complicated_oral	1664	f	Low trimethoprim resistance	specificity.c	0.85	uniform	0.75	1	specificity to that antibiotic - current	Assumption	cefalexin
complicated_oral	1664	f	Low trimethoprim resistance	cost.empiricuti.U	2.41	uniform	1.49	3.19	cost of empiric therapy (one off)	British National Formulary (20) Cost per tab into cost per course (21–25)	cefalexin
complicated_oral	1664	f	Low trimethoprim resistance	cost.empiricuti.current	0.57	uniform	0.35	0.75	cost of empiric therapy for those that then switch	British National Formulary (20) Cost per tab into cost per course (21–25)	cefalexin

complicated_oral	1664	f	Low trimethoprim resistance	cost.empiricuti.ifast	0.11	uniform	0.07	0.15	cost of empiric therapy for those that then switch	British National Formulary (20) Cost per tab into cost per course (21–25)	cefalexin
complicated_oral	1664	f	Low trimethoprim resistance	cost.2ndline.U	4.06	uniform	3.32	13.53	cost of second line (one off)	British National Formulary (20) Cost per tab into cost per course (21–25)	trimethoprim
complicated_oral	65100	f	Low trimethoprim resistance	r.prev	0.13	uniform	0.08	0.18	prevalence of that resistance profile	Assumption. Estimates in (16,18)	cefalexin
complicated_oral	65100	f	Low trimethoprim resistance	sensitivity.i	0.95	uniform	0.75	1	sensitivity to that antibiotic - BICT (iFAST)	Assumption	cefalexin
complicated_oral	65100	f	Low trimethoprim resistance	specificity.i	0.85	uniform	0.75	1	specificity to that antibiotic - BICT (iFAST)	Assumption	cefalexin
complicated_oral	65100	f	Low trimethoprim resistance	sensitivity.c	0.95	uniform	0.75	1	sensitivity to that antibiotic - current	Assumption	cefalexin
complicated_oral	65100	f	Low trimethoprim resistance	specificity.c	0.85	uniform	0.75	1	specificity to that antibiotic - current	Assumption	cefalexin
complicated_oral	65100	f	Low trimethoprim resistance	cost.empiricuti.U	2.41	uniform	1.49	3.19	cost of empiric therapy (one off)	British National Formulary (20) Cost per tab into cost per course (21–25)	cefalexin
complicated_oral	65100	f	Low trimethoprim resistance	cost.empiricuti.current	0.57	uniform	0.35	0.75	cost of empiric therapy for those that then switch	British National Formulary (20) Cost per tab into cost per course (21–25)	cefalexin
complicated_oral	65100	f	Low trimethoprim resistance	cost.empiricuti.ifast	0.11	uniform	0.07	0.15	cost of empiric therapy for those that then switch	British National Formulary (20) Cost per tab into cost per course (21–25)	cefalexin
complicated_oral	65100	f	Low trimethoprim resistance	cost.2ndline.U	4.06	uniform	3.32	13.53	cost of second line (one off)	British National Formulary (20) Cost per tab into cost per course (21–25)	trimethoprim
complicated_oral	1664	m	Low trimethoprim resistance	r.prev	0.13	uniform	0.08	0.18	prevalence of that resistance profile	Assumption. Estimates in (16,18)	cefalexin
complicated_oral	1664	m	Low trimethoprim resistance	sensitivity.i	0.95	uniform	0.75	1	sensitivity to that antibiotic - BICT (iFAST)	Assumption	cefalexin
complicated_oral	1664	m	Low trimethoprim resistance	specificity.i	0.85	uniform	0.75	1	specificity to that antibiotic - BICT (iFAST)	Assumption	cefalexin

complicated_oral	1664	m	Low trimethoprim resistance	sensitivity.c	0.95	uniform	0.75	1	sensitivity to that antibiotic - current	Assumption	cefalexin
complicated_oral	1664	m	Low trimethoprim resistance	specificity.c	0.85	uniform	0.75	1	specificity to that antibiotic - current	Assumption	cefalexin
complicated_oral	1664	m	Low trimethoprim resistance	cost.empiricuti.U	2.41	uniform	1.49	3.19	cost of empiric therapy (one off)	British National Formulary (20)<br>Cost per tab into cost per course (21–25)	cefalexin
complicated_oral	1664	m	Low trimethoprim resistance	cost.empiricuti.current	0.57	uniform	0.35	0.75	cost of empiric therapy for those that then switch	British National Formulary (20)<br>Cost per tab into cost per course (21–25)	cefalexin
complicated_oral	1664	m	Low trimethoprim resistance	cost.empiricuti.ifast	0.11	uniform	0.07	0.15	cost of empiric therapy for those that then switch	British National Formulary (20)<br>Cost per tab into cost per course (21–25)	cefalexin
complicated_oral	1664	m	Low trimethoprim resistance	cost.2ndline.U	4.06	uniform	3.32	13.53	cost of second line (one off)	British National Formulary (20)<br>Cost per tab into cost per course (21–25)	trimethoprim
complicated_oral	65100	m	Low trimethoprim resistance	r.prev	0.13	uniform	0.08	0.18	prevalence of that resistance profile	Assumption. Estimates in (16,18)	cefalexin
complicated_oral	65100	m	Low trimethoprim resistance	sensitivity.i	0.95	uniform	0.75	1	sensitivity to that antibiotic - BICT (iFAST)	Assumption	cefalexin
complicated_oral	65100	m	Low trimethoprim resistance	specificity.i	0.85	uniform	0.75	1	specificity to that antibiotic - BICT (iFAST)	Assumption	cefalexin
complicated_oral	65100	m	Low trimethoprim resistance	sensitivity.c	0.95	uniform	0.75	1	sensitivity to that antibiotic - current	Assumption	cefalexin
complicated_oral	65100	m	Low trimethoprim resistance	specificity.c	0.85	uniform	0.75	1	specificity to that antibiotic - current	Assumption	cefalexin
complicated_oral	65100	m	Low trimethoprim resistance	cost.empiricuti.U	2.41	uniform	1.49	3.19	cost of empiric therapy (one off)	British National Formulary (20)<br>Cost per tab into cost per course (21–25)	cefalexin
complicated_oral	65100	m	Low trimethoprim resistance	cost.empiricuti.current	0.57	uniform	0.35	0.75	cost of empiric therapy for those that then switch	British National Formulary (20)<br>Cost per tab into cost per course (21–25)	cefalexin
complicated_oral	65100	m	Low trimethoprim resistance	cost.empiricuti.ifast	0.11	uniform	0.07	0.15	cost of empiric therapy for those that then switch	British National Formulary (20)<br>Cost per tab into cost per	cefalexin

										course (21–25)	
complicated_oral	65100	m	Low trimethoprim resistance	cost.2ndline.U	4.06	uniform	3.32	13.53	cost of second line (one off)	British National Formulary (20) Cost per tab into cost per course (21–25)	trimethoprim
complicated_oral	1664	f	all	cohort	3221.1				Total in that cohort	Calculated from HES data (14)	
complicated_oral	65100	f	all	cohort	11932.7				Total in that cohort	Calculated from HES data (14)	
complicated_oral	1664	m	all	cohort	1719.2				Total in that cohort	Calculated from HES data (14)	
complicated_oral	65100	m	all	cohort	7766.5				Total in that cohort	Calculated from HES data (14)	

Table 3: Cohort sizes used within the model.

UTI type	Age group	Sex	Cohort size
Uncomplicated	16-64	F	25769
Complicated IV	16-64	F	3221
Uncomplicated	65-100	F	95462
Complicated IV	65-100	F	11933

Uncomplicated	16-64	M	13754
Complicated IV	16-64	M	1719
Uncomplicated	65-100	M	62132
Complicated IV	65-100	M	7767
Complicated oral	16-64	F	3221
Complicated oral	65-100	F	11933
Complicated oral	16-64	M	1719
Complicated oral	65-100	M	7767

Figure 2: Bed days saved across age and sex groups and complicated IV, complicated oral and uncomplicated UTI.

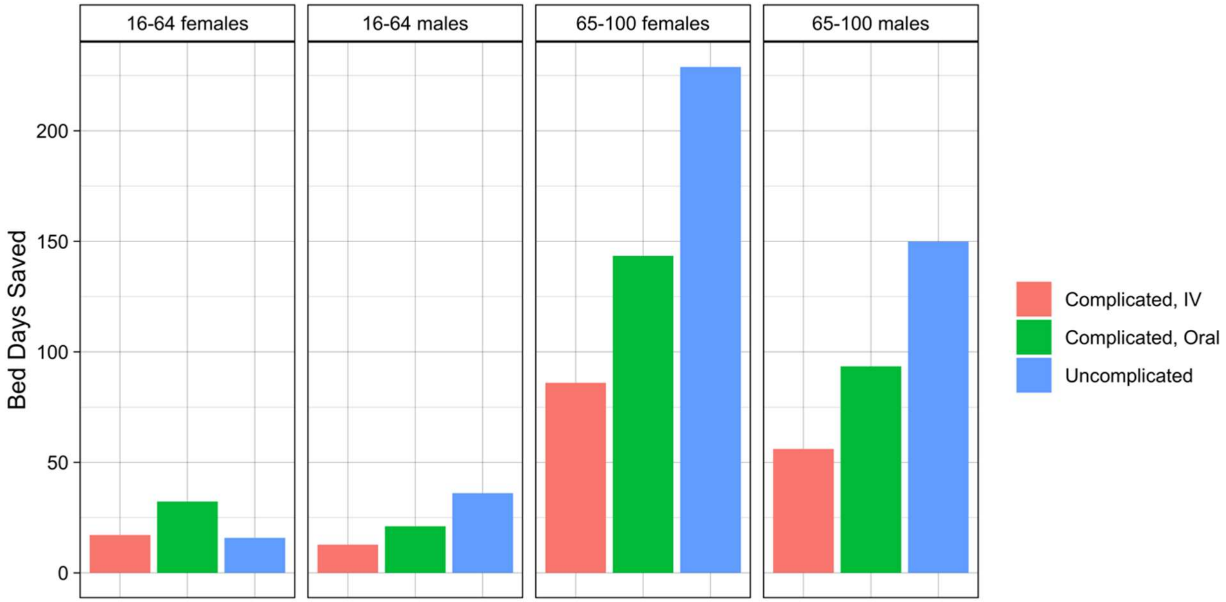

Figure 3: Incremental cost, incremental QALY gain, and net monetary benefit for each population level scenario.

This is split into type of UTI and age and sex groups

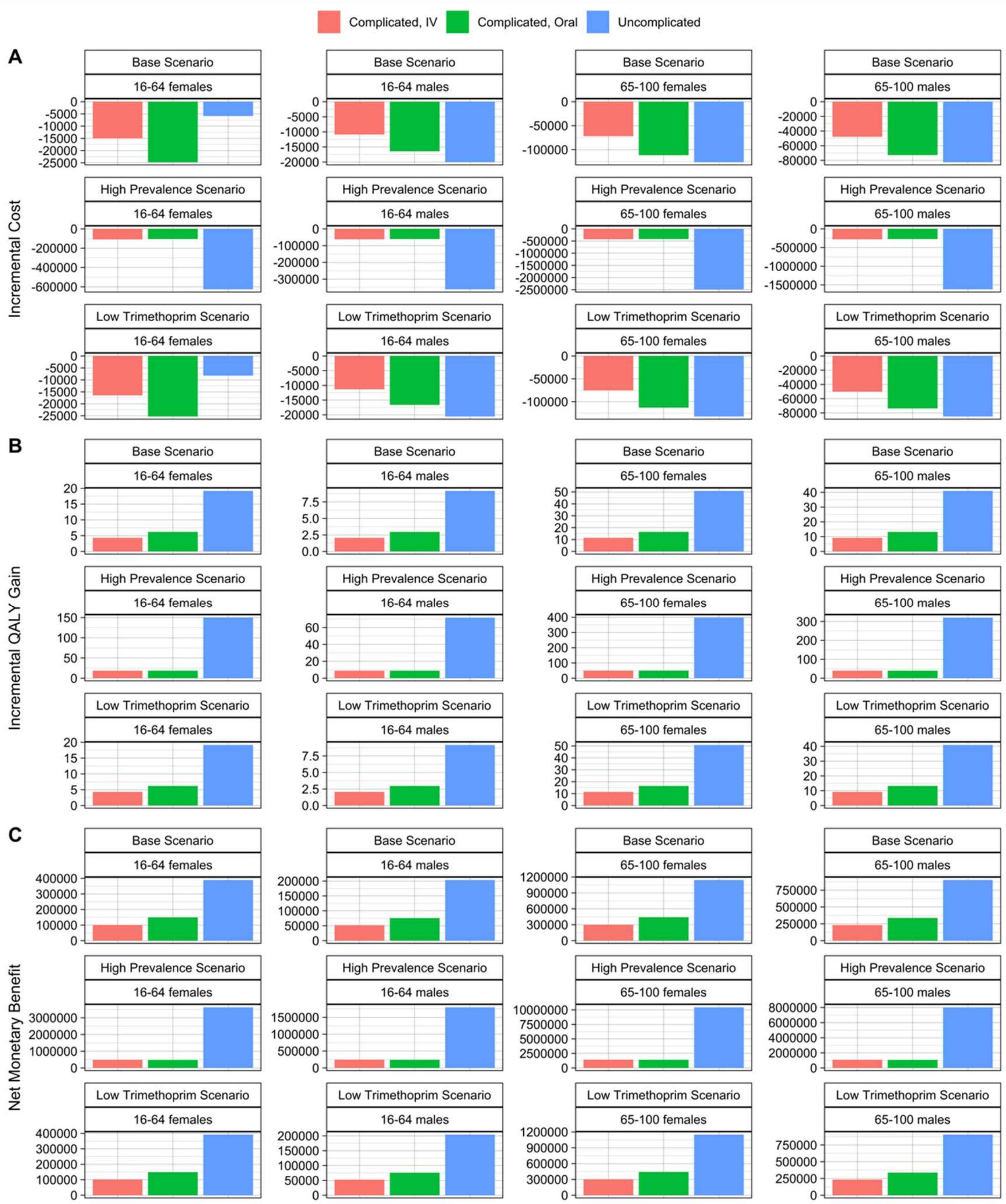

Figure 4: Turnaround time for BICT and cost of BICT Sensitivity Analyses

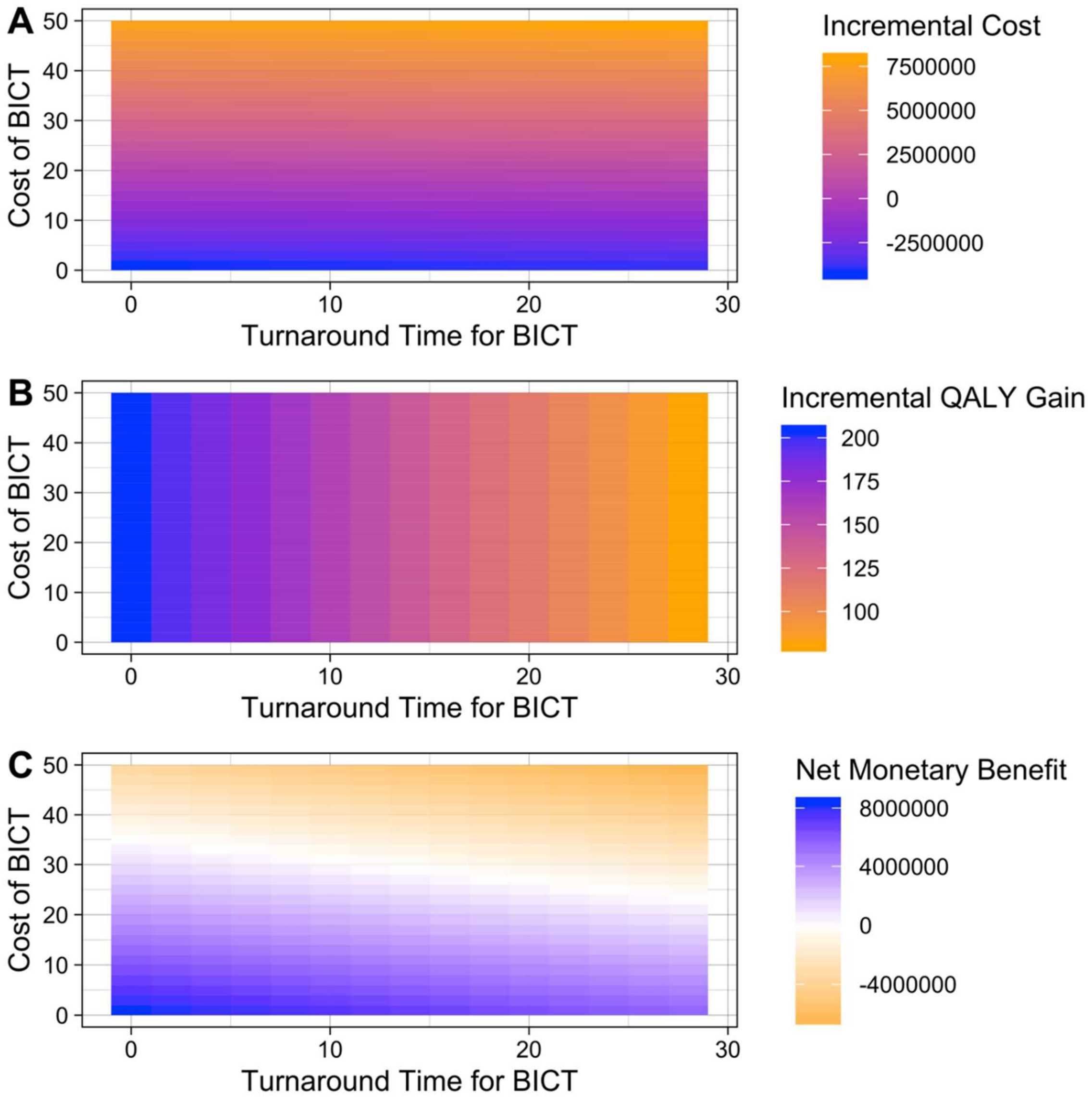

Figure 5A: Univariate sensitivity analysis for all parameters in the model for the outcome of Net Monetary Benefit.

All descriptions of parameters can be found in Table S2: full parameter table.

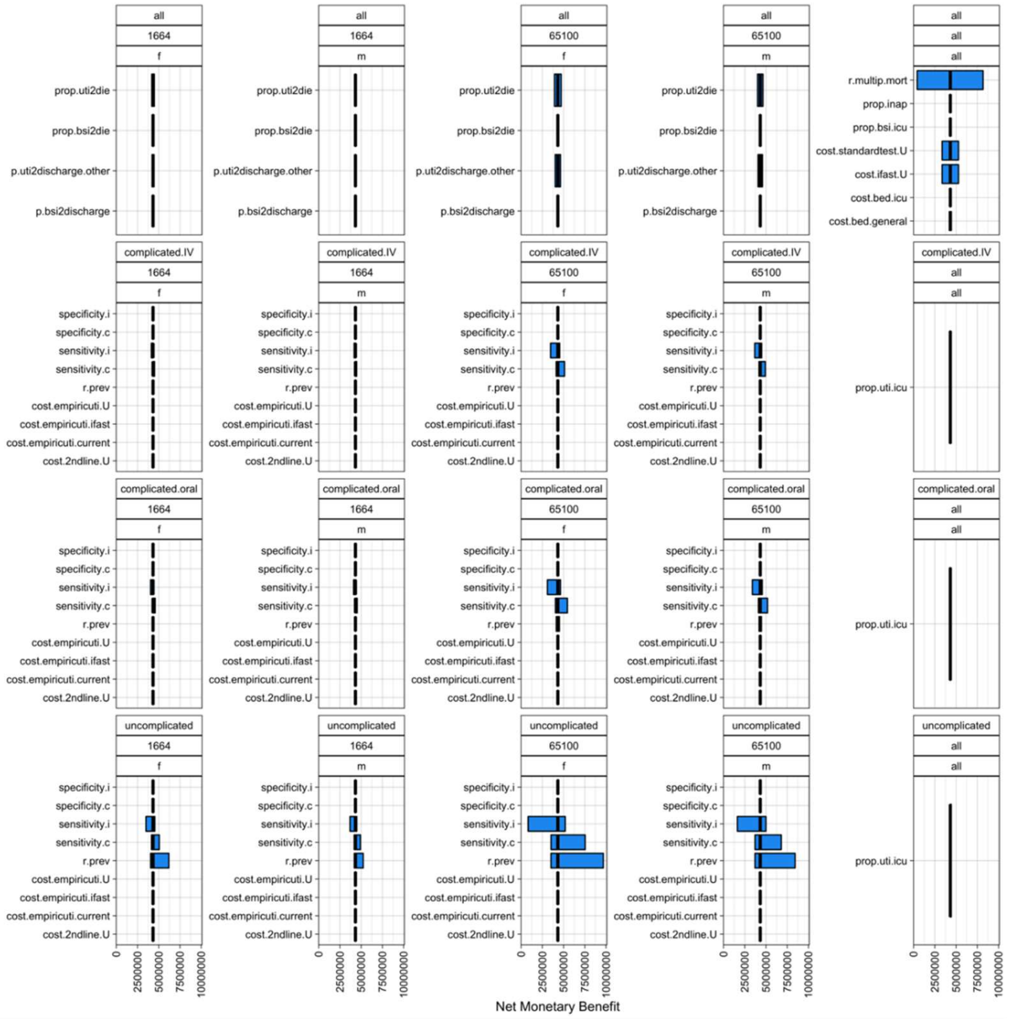

Figure 5B: Univariate sensitivity analysis for all parameters in the model for the outcome of Incremental Cost.

All descriptions of parameters can be found in Table S2: full parameter table.

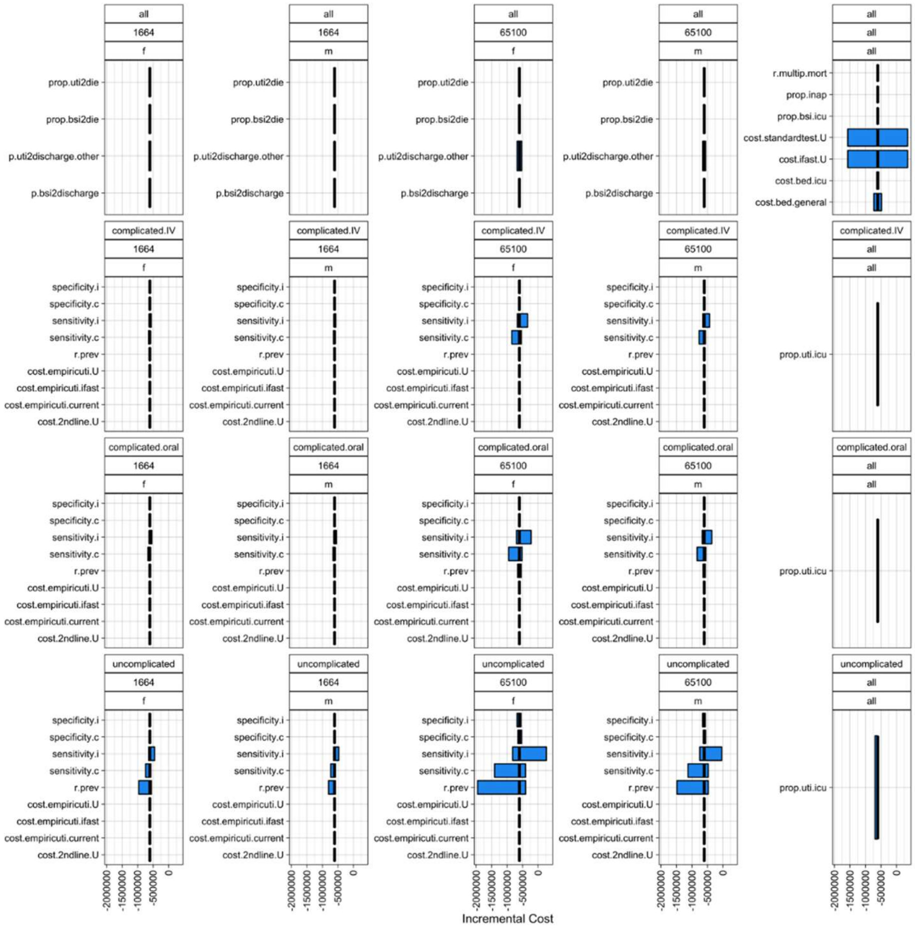

Figure 5C: Univariate sensitivity analysis for all parameters in the model for the outcome of Incremental QALYs.
All descriptions of parameters can be found in Table S2: full parameter table.

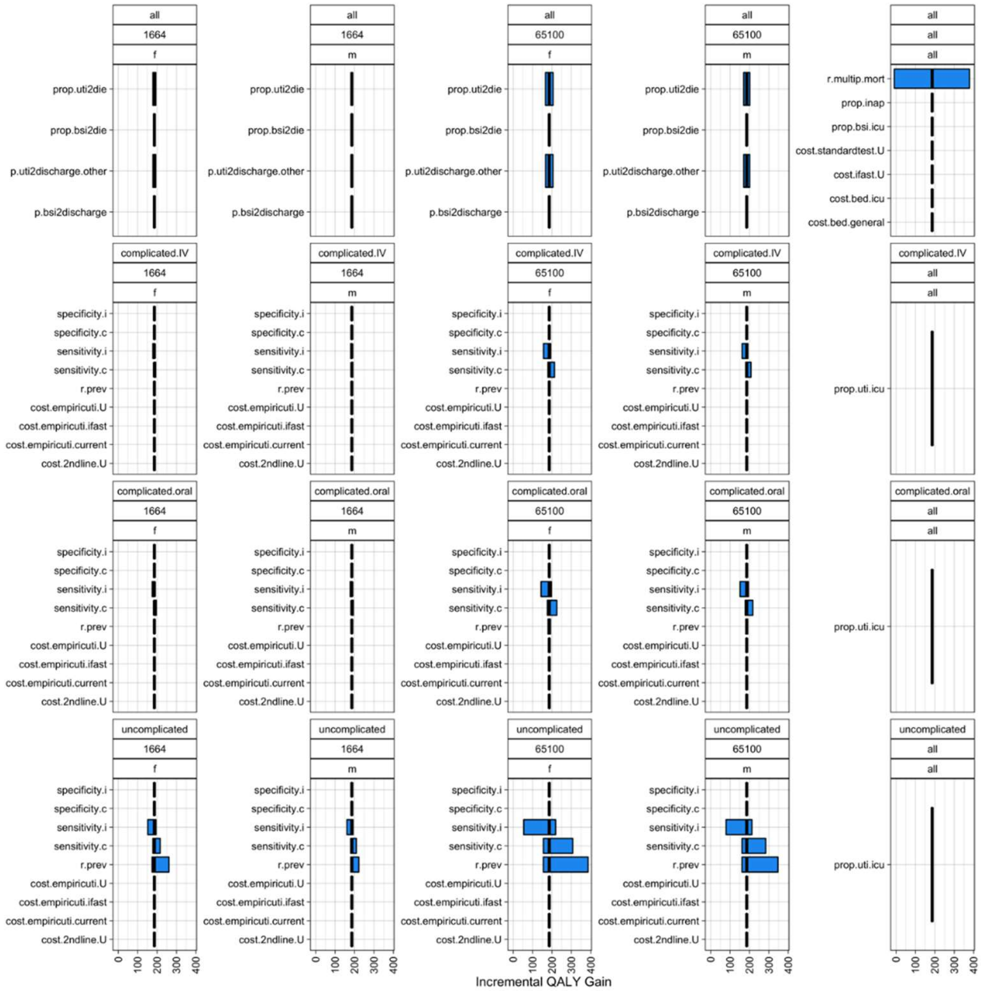

Table 4. CHEERS Checklist

From: Husereau D, Drummond M, Augustovski F, et al. Consolidated Health Economic Evaluation Reporting Standards 2022 (CHEERS 2022) Explanation and Elaboration: A Report of the ISPOR CHEERS II Good Practices Task Force. Value Health 2022;25. doi:10.1016/j.jval.2021.10.008

Topic	No.	Item	Location where item is reported
Title			
	1	Identify the study as an economic evaluation and specify the interventions being compared.	Title, Page 1
Abstract			
	2	Provide a structured summary that highlights context, key methods, results, and alternative analyses.	Abstract, Page 2
Introduction			Pg5
Background and objectives	3	Give the context for the study, the study question, and its practical relevance for decision making in policy or practice.	Introduction, page 5
Methods			Pg 7
Health economic analysis plan	4	Indicate whether a health economic analysis plan was developed and where available.	Not Applicable
Study population	5	Describe characteristics of the study population (such as age range, demographics, socioeconomic, or clinical characteristics).	Methods, page 7-13, & Supplementary Table 3
Setting and location	6	Provide relevant contextual information that may influence findings.	Methods, page 7-13 & Supplementary Material Figure 1
Comparators	7	Describe the interventions or strategies being compared and why chosen.	Methods, page 10,13
Perspective	8	State the perspective(s) adopted by the study and why chosen.	Methods, page 7 & Box 1
Time horizon	9	State the time horizon for the study and why appropriate.	Methods, page 9

Topic	No.	Item	Location where item is reported
Discount rate	10	Report the discount rate(s) and reason chosen.	Methods, page 9
Selection of outcomes	11	Describe what outcomes were used as the measure(s) of benefit(s) and harm(s).	Methods, page 10,14
Measurement of outcomes	12	Describe how outcomes used to capture benefit(s) and harm(s) were measured.	Methods, page 14
Valuation of outcomes	13	Describe the population and methods used to measure and value outcomes.	Methods, page 14
Measurement and valuation of resources and costs	14	Describe how costs were valued.	Methods, page 12-14
Currency, price date, and conversion	15	Report the dates of the estimated resource quantities and unit costs, plus the currency and year of conversion.	Methods, page 10,11
Rationale and description of model	16	If modelling is used, describe in detail and why used. Report if the model is publicly available and where it can be accessed.	Methods, pages 7 – 14
Analytics and assumptions	17	Describe any methods for analysing or statistically transforming data, any extrapolation methods, and approaches for validating any model used.	Methods, pages 12 – 16
Characterising heterogeneity	18	Describe any methods used for estimating how the results of the study vary for subgroups.	Methods , page 13-15
Characterising distributional effects	19	Describe how impacts are distributed across different individuals or adjustments made to reflect priority populations.	Methods , page 14-15
Characterising uncertainty	20	Describe methods to characterise any sources of uncertainty in the analysis.	Methods, page 10
Approach to engagement with patients and others affected by the study	21	Describe any approaches to engage patients or service recipients, the general public, communities, or stakeholders (such as clinicians or payers) in the design of the study.	Acknowledgements, page 1 & Methods, Patient & Public involvement section
Results			
Study parameters	22	Report all analytic inputs (such as values, ranges, references) including uncertainty or distributional assumptions.	Methods, Table 1
Summary of main results	23	Report the mean values for the main categories of costs and outcomes of interest and summarise them in the most appropriate overall measure.	Results, Table 2 – 3

Topic	No.	Item	Location where item is reported
Effect of uncertainty	24	Describe how uncertainty about analytic judgments, inputs, or projections affect findings. Report the effect of choice of discount rate and time horizon, if applicable.	Results, Probabilistic Sensitivity Analysis section, page 17 & 18
Effect of engagement with patients and others affected by the study	25	Report on any difference patient/service recipient, general public, community, or stakeholder involvement made to the approach or findings of the study	Not Applicable
Discussion			
Study findings, limitations, generalisability, and current knowledge	26	Report key findings, limitations, ethical or equity considerations not captured, and how these could affect patients, policy, or practice.	Discussion, pages 19-23
Other relevant information			
Source of funding	27	Describe how the study was funded and any role of the funder in the identification, design, conduct, and reporting of the analysis	Page 24
Conflicts of interest	28	Report authors conflicts of interest according to journal or International Committee of Medical Journal Editors requirements.	Page 24

Table 5: Total inappropriate and appropriate prescribing-days of antibiotics under current baseline and BICT

	Bed Days	BSI days	Inappropriate Prescribing Days for Resistant Infections
Current	4,659,754	7557	43,204
BICT	4,658,861	7554	18,571
Decrease due to BICT	893	3	24,632
Percentage Decrease	0.02%	0.04%	57%

Table 6. Deterministic Base Results by Population Group

	Scenario	Age Group	Sex	Excess Cost	Excess QALY Loss	Incremental Cost	Incremental QALY Gain	Net Monetary Benefit
Current	Uncomplicated	16-64	F	200827029	11029.3962	NA	NA	NA
BICT	Uncomplicated	16-64	F	200821156	11010.2196	-5872.9259	19.1766473	389405.871
Current	Uncomplicated	16-64	M	170203357	8168.53723	NA	NA	NA
BICT	Uncomplicated	16-64	M	170183292	8159.36205	-20064.916	9.17518261	203568.568
Current	Uncomplicated	65-100	F	1109542859	42638.2705	NA	NA	NA
BICT	Uncomplicated	65-100	F	1109416509	42587.4334	-126349.25	50.8370555	1143090.36
Current	Uncomplicated	65-100	M	725185236	34473.6575	NA	NA	NA
BICT	Uncomplicated	65-100	M	725102683	34432.6837	-82553.116	40.9737854	902028.823
Current	Complicated IV	16-64	F	33783161.6	1377.02682	NA	NA	NA

BICT	Complicated IV	16-64	F	33768104	1372.72332	-15057.547	4.30349898	101127.527
Current	Complicated IV	16-64	M	28592062.8	1019.29547	NA	NA	NA
BICT	Complicated IV	16-64	M	28581194.8	1017.23639	-10867.974	2.05908042	52049.582
Current	Complicated IV	65-100	F	186385765	5320.85556	NA	NA	NA
BICT	Complicated IV	65-100	F	186313836	5309.44682	-71929.417	11.4087451	300104.32
Current	Complicated IV	65-100	M	121851561	4301.96711	NA	NA	NA
BICT	Complicated IV	65-100	M	121803660	4292.77186	-47901.376	9.19524697	231806.316
Current	Complicated Oral	16-64	F	33631542.6	1375.39325	NA	NA	NA
BICT	Complicated Oral	16-64	F	33606771.5	1369.19322	-24771.107	6.20003462	148771.799
Current	Complicated Oral	16-64	M	28490873.3	1017.53714	NA	NA	NA

BICT	Complicated Oral	16-64	M	28474432.5	1014.57057	-16440.886	2.96657488	75772.3841
Current	Complicated Oral	65-100	F	185788856	5311.99539	NA	NA	NA
BICT	Complicated Oral	65-100	F	185677264	5295.55854	-111592.59	16.4368567	440329.721
Current	Complicated Oral	65-100	M	121398748	4294.7822	NA	NA	NA
BICT	Complicated Oral	65-100	M	121326011	4281.53439	-72737.037	13.2478109	337693.254

References

1. NICE. NICE Methods Manual. 2014;(October).
2. Harvard T.H. Chan School of Public Health. https://chds.hsph.harvard.edu/approaches/cost-effectiveness-analysis/. 2024. Cost-Effectiveness Analysis.
3. NICE. Preoperative tests: Routine preoperative tests for elective surgery. 2015.
4. CPI Inflation Calculator. U.K. Inflation Calculator [Internet]. 2022. Available from: https://www.officialdata.org/UK-inflation
5. Guest JF, Keating T, Gould D, Wigglesworth N. Modelling the annual NHS costs and outcomes attributable to healthcare-associated infections in England. BMJ Open. 2020;10(1):e033367.
6. Eliakim-Raz N, Babitch T, Shaw E, Addy I, Wiegand I, Vank C, et al. Risk factors for treatment failure and mortality among hospitalized patients with complicated urinary tract infection: A multicenter retrospective cohort study (RESCUING study group). Clinical Infectious Diseases. 2019;68(1).
7. Holmbom M, Andersson M, Berg S, Eklund D, Sobczynski P, Wilhelms D, et al. Prehospital delay is an important risk factor for mortality in community-acquired bloodstream infection (CA-BSI): A matched case-control study. BMJ Open. 2021;11(11).

8. Naylor NR. The burden of antimicrobial resistance: the case of escherichia coli. 2019.
9. Shallcross L, Rockenschaub P, Blackburn R, Nazareth I, Freemantle N, Hayward A. Antibiotic prescribing for lower UTI in elderly patients in primary care and risk of bloodstream infection: A cohort study using electronic health records in England. PLoS Med. 2020;17(9):e1003336.
10. Wozniak TM, Dyda A, Lee X. The Increased Length of Hospital Stay and Mortality Associated With Community-Associated Infections in Australia. Open Forum Infect Dis. 2022;9(5):ofac133.
11. Public Health England. Research reveals levels of inappropriate prescriptions in England [Internet]. 2018. Available from: https://www.gov.uk/government/news/research-reveals-levels-of-inappropriate-prescriptions-in-england
12. Shafrin J, Marijam A, Joshi A V., Mitrani-Gold FS, Everson K, Tuly R, et al. Impact of suboptimal or inappropriate treatment on healthcare resource use and cost among patients with uncomplicated urinary tract infection: an analysis of integrated delivery network electronic health records. Antimicrob Resist Infect Control. 2022;11(1).
13. Naylor NR. The COVID-19 QALY Loss Calculator for Associated Deaths. 2020.
14. NHS Digital. Hospital Episode Statistics (HES) data. 2022.
15. Kahlmeter G, Åhman J, Matuschek E. Antimicrobial resistance of Escherichia coli causing uncomplicated urinary tract infections: a European update for 2014 and comparison with 2000 and 2008. Infect Dis Ther. 2015;4:417–23.
16. Baines G, Banjoko A, Brair A, Gray J, Desai N, Cardozo L, et al. Antibiotic resistance in urinary tract infections: A re-visit after five years and experience over two sites. Post Reprod Health. 202AD;26(2):91–100.
17. Tutone M, Johansen TEB, Cai T, Mushtaq S, Livermore DM. Susceptibility and Resistance to Fosfomycin and other antimicrobial agents among pathogens causing lower urinary tract infections: findings of the SURF study. Int J Antimicrob Agents. 2022;59(5):106574.
18. Toner L et al. Extended-spectrum beta-lactamase-producing Enterobacteriaceae in hospital urinary tract infections: incidence and antibiotic susceptibility profile over 9 years. World J Urol. 2016;24:1031–7.
19. Somorin YM, Weir NJM, Pattison SH, Crockard MA, Hughes CM, Tunney MM, et al. Antimicrobial resistance in urinary pathogens and culture-independent detection of trimethoprim resistance in urine from patients with urinary tract infection. BMC Microbiol. 2022;22(1):1–8.
20. NICE. BNF British National Formulary. 2023.
21. NICE. NICE Guideline NG113. 2018. Urinary tract infection (catheter-associated): antimicrobial prescribing. Available from: https://www.nice.org.uk/guidance/ng113
22. NICE. NICE Guideline NG109. 2018. Urinary tract infection (lower): antimicrobial prescribing. Available from: https://www.nice.org.uk/guidance/ng109
23. NICE. NICE Guideline NG112. 2018. Urinary tract infection (recurrent): antimicrobial prescribing. Available from: https://www.nice.org.uk/guidance/ng112
24. North Bristol NHS Trust. Antibiotic Guidelines. 2020.

25. Essex Partnership University NHS Foundation Trust. Section 18: Antimicrobial Prescribing. 2022.
