# Supplementary material for: Rapid antibiotic susceptibility testing for urinary tract infections in secondary care in England: a cost-effectiveness analysis
Source: BMJ Open. 2024 Nov 28;14(11):e081865. doi: 10.1136/bmjopen-2023-081865 (PMC11867666; doi:10.1136/bmjopen-2023-081865)
Supplement: Supplementary file 2 [file bmjopen-2023-081865.reviewer_comments.pdf]

PEER REVIEW HISTORY

BMJ Open publishes all reviews undertaken for accepted manuscripts. Reviewers are asked to complete a checklist review form and are provided with free text boxes to elaborate on their assessment. These free text comments are reproduced below.

ARTICLE DETAILS

Title (Provisional)

RAPID ANTIBIOTIC SUSCEPTIBILITY TESTING FOR URINARY TRACT INFECTIONS IN SECONDARY CARE IN ENGLAND: A COST-EFFECTIVENESS ANALYSIS

Authors

Booton, Ross D; Agnew, Emily; Pople, Diane; Evans, Stephanie; Bock, Lucy J.; Sutton, J. Mark; Robotham, Julie; Naylor, Nichola R

VERSION 1 - REVIEW

**Reviewer**	**1**
**Name**	**Stewart, Sarah-Jane F.**
**Affiliation**	**University College London, Research department of practice and policy, School of Pharmacy**
**Date**	**13-Dec-2023**
**COI**	**None.**

You address an important and highly topical area of research in this paper, highlighting the potential cost-utility from an NHS system perspective of the BICT.

Your manuscript is clearly organised and easy to follow. You have used figures appropriately, acknowledged the limitations of your approach and drawn suitable conclusions from the data you present.

**Reviewer**	**2**
**Name**	**Ramírez-Sevilla , Christobal**
**Affiliation**	**Hospital Sant Joan de Deu, UROLOGY**
**Date**	**14-Dec-2023**
**COI**	**I don't have competing interests.**

The study carried out on 280,000 patients was very extensive. This means that the sample size was very representative.

The statistics, figures and tables are very extensive and representative, congratulations.

The study is very interesting because it refers to the economic savings and improvement in the quality of life of patients with urinary infections throughout an entire country, but needs some important details to be reviewed to improve the quality of work and improve the quality of scientific knowledge.

The references are adequate in number.

Consent for publication is not provided.

Consent to participate of all authors is not included.

Ethics approval: why in a study of 280,000 patients the ethics statement was considered not applicable? The reasoning should be included in the manuscript.

Corresponding author is missing.

Whether or not written informed consent was obtained from all participants should be included in the manuscript.

List of abbreviations is not provided.

List of contributions authors to the study is not included.

Another limitation of this work is that it is a retrospective study. This should be added to the "Strengths and limitations of the study" section.

The definition of the Markov model should be included in the manuscript to clarify the concept.

In the materials and methods section it is advisable not to include references, which correspond to the discussion. Except for some that are very essential.

Box-1 should not be included in the manuscript, but at the end with figures and other tables.

All figures and tables must be referenced in the manuscript and arranged by number in order of appearance.

Tables 1, 2, 3 and 4 should not be included in the manuscript, but at the end with the figures and other tables.

In the manuscript after the introduction the aim of the study should be included separately.

On line 300, the sentence "...a cohort size of 1..." is confusing, it should be explained more clearly.

At the end of the manuscript, conclusions and recommendations section must be included separately.

References 5, 6, 8, 17, 27, 30, 32, 33, 38 and 29 are incomplete due to lack of pages.

Reference 15 is not indexed and difficult to find, in my opinion it should be removed from the manuscript.

**** Reviewer comments.docx ****

**Reviewer**	**3**
**Name**	**Bao, Chang-jun**
**Affiliation**	**Jiangsu Provincial Center for Disease Control and Prevention, Acute Infectious Disease Control and Prevention**
**Date**	**16-Jan-2024**
**COI**	**I have no competing interests here.**

The manuscript intends to analysis the cost-effectiveness of rapid antibiotic susceptibility testing for urinary tract infection based on Markov model. A few minor issues need to be addressed before it is accepted for publication.

Please explain the rationality of using Markov model to analysis.

Table 1, Line 289: What are the considerations behind the consistency of culture and BICT sensitivity and specificity? Is there any literature to support it? In general, the sensitivity and specificity of rapid assessment will not be consistent with the culture results.

Line 296-297: Why choose the data for the three years 2017-2019 as the cohort instead of the three-year average?

Line 488: The transition between the stars and the health states is not very clear, for example, what is the relationship between the BSI and the subsequent treat state?

Line 500, Figure 3: From Figure 3, only the patients with uncomplicated UTI gained more obvious benefits compared to the patients with complicated IV or Oral. Did it indicated that the impedance-based fast antimicrobial susceptibly test (iFAST) using bacterial impedance cytometry technology(BICT) has great limitations?

**Reviewer**	**4**
**Name**	**Parmar, Priya**
**Affiliation**	**Auckland University of Technology**
**Date**	**22-Feb-2024**
**COI**	**N/A**

Great sample size for the administrative dataset – excellent resource.

Why are the transition probabilities not all from the dataset collected (size which appears substantially large) I.e. Lines 256-278 state that transition probabilities from UTI to BSI to discharge and death are estimated using the hospital administrative data whereas transition probabilities for susceptible UTIs and UTI to BSI rates from literature and ICU vs. general ward. After looking at Supplementary Table S2 (which is well-detailed and describes for which transition probability was calculated and which was from said published resource). It would be useful to state in the legend of this table, or in the main methods section justification for why published literature was used vs. administrative data calculations (happy that it has been done this way just want it to be clarified and explained for the reader). For those where there has been a calculation can those equations be added to Supplementary Table S2. This will allow for higher reproducibility of results from peer researchers (like the sources column in Table 1).

Have any of the transition probabilities been generated using a priori estimates from the published literature and cascades from other calculated transition probabilities (as listed in Supplementary Table S2) for the Markov Model?

Compared to 'Cost of standard test' which had the original and then inflated costs stated in Table 1, it was not clear if this was the case for 'Hourly cost of a general bed day' and 'Hourly cost of an ICU bed day'.

**Reviewer**	**5**
**Name**	**Garbett, Shawn**
**Affiliation**	**Vanderbilt University Medical Center, Biostatistics**
**Date**	**01-Mar-2024**
**COI**	**None**

Page 51.4 supplementary misspelled.

Overall this paper was a pleasure to read, really well written and laid out with clear results. This is a first for me as a reviewer. I think it's an important result that needs to be published and a fine piece of work.

Minor comment. One thing I was not entirely convinced on was the simple claim that the results were conservative under the limitation of not having an epidemiology (dynamic transmission) model. There's two dimensions: the subsequent transmission rates resulting in an overall change in population affected (knock-on costs) and the population mixture of infection types. Knock-on costs are a total cost to a health system, thus in this context the results are conservative. However, the additional dimension of changing the mixture of infection types could work against being a conservative estimate. The boundary case would the elimination of the problematic infections in the population, in which case the cost of

testing would be of no benefit and the method would not be cost effective. Such a boundary case is implausible. Additionally having such testing available could have potential benefits for other disease processes. Thus the limitation has more nuance than stated and likely still leans towards conservative when everything is considered. Given the overall strength of the results I view this as a very minor thing.

VERSION 1 - AUTHOR RESPONSE

Dear Dr Zainab Yusuf,

On behalf of my co-authors, I would like to thank you for the opportunity to revise and resubmit our manuscript "Rapid Antibiotic Susceptibility Testing for Urinary Tract Infections in Secondary Care in England: A cost-effectiveness analysis " (bmjopen-2023-081865) to BMJ Open.

Thank you for providing the reviewers' and Editorial comments, we have adjusted our manuscript accordingly, resulting in a much clearer manuscript. We have provided a `tracked changes'/marked version and a clean version of the manuscript and supplementary material. Additionally, as part of our resubmission we have included a detailed, point-by-point response to the various comments by yourself and the reviewers.

We have also now conducted the more recent edits requested "prior to Editor assignment" to the clean copy of the manuscript, which is reuploaded, with individual responses also available in the response document.

This study highlights, for the first time, the health and cost benefits of a rapid diagnostic for antibiotic susceptibility of urinary tract infections in NHS hospitals. Such evidence can help inform policy in the treatment of UTI patients, and in the fight against antibiotic resistance. This is timely work given the recent development of a rapid UTI diagnostic receiving the Longitude Prize.

We hope that the revised manuscript now meets the journal's high standards and we look forward to hearing from you.

Yours sincerely,

Dr Nichola Naylor (on behalf of all co-authors)

VERSION 2 - REVIEW

Reviewer 3

Name Bao, Chang-jun

Affiliation Jiangsu Provincial Center for Disease Control and Prevention, Acute Infectious Disease Control and Prevention

Date 12-Aug-2024

COI I have no competing interests here.

I am satisfied with the authors' answer.

Reviewer 5

Name Garbett, Shawn

Affiliation Vanderbilt University Medical Center, Biostatistics

Date 02-Aug-2024

COI I have no competing interests.

The authors have dealt with all feedback. On a second read through, I think the PSA indirectly deals with some of the possible effects of downstream epidemiology effects.
